# Control of Multilayer Networks

**DOI:** 10.1038/srep20706

**Published:** 2016-02-12

**Authors:** Giulia Menichetti, Luca Dall’Asta, Ginestra Bianconi

**Affiliations:** 1 Department of Physics and Astronomy and INFN Sez. Bologna, Bologna University, Viale B. Pichat 6/2 40127 Bologna, Italy; 2 Department of Applied Science and Technology, DISAT, Politecnico di Torino, Corso Duca degli Abruzzi 24, 10129 Torino, Italy; 3 Collegio Carlo Alberto, Via Real Collegio 30, 10024 Moncalieri, Italy; 4 School of Mathematical Sciences, Queen Mary University of London, London E1 4NS, United Kingdom

## Abstract

The controllability of a network is a theoretical problem of relevance in a variety of contexts ranging from financial markets to the brain. Until now, network controllability has been characterized only on isolated networks, while the vast majority of complex systems are formed by multilayer networks. Here we build a theoretical framework for the linear controllability of multilayer networks by mapping the problem into a combinatorial matching problem. We found that correlating the external signals in the different layers can significantly reduce the multiplex network robustness to node removal, as it can be seen in conjunction with a hybrid phase transition occurring in interacting Poisson networks. Moreover we observe that multilayer networks can stabilize the fully controllable multiplex network configuration that can be stable also when the full controllability of the single network is not stable.

Most of the real networks are not isolated but interact with each other forming multilayer structures^1^,^2^. For example, banks are linked to each other by different types of contracts and relationships, gene regulation in the cell is mediated by the different types of interactions between different kinds of molecules, brain data are described by multilayer brain networks. Studying the controllability properties of these networks is important for assessing the risk of a financial crash^3^,^4^, for drug discovery^5^ and for characterizing brain dynamics^6^,^7^,^8^,^9^,^10^. Therefore the controllability of multilayer networks is a problem of fundamental importance for a large variety of applications.

Recently, linear^11^,^12^,^13^,^14^,^15^,^16^,^17^,^18^,^19^ and non-linear^20^,^21^,^22^,^23^,^24^,^25^,^26^,^27^,^28^,^29^,^30^ approaches are providing new scenarios for the characterization of the controllability of single complex networks. In particular, in the seminal paper by Liu *et al.*^12^ the structural controllability of complex networks has been addressed by mapping this problem into a Maximum Matching Problem that can be studied using statistical mechanics techniques^31^,^32^,^33^,^34^,^35^,^36^. Other works approach the related problem of network observability^37^, or target control^38^ which focuses on controlling just a subset of the nodes. Despite the significant interest in network controllability, all linear and non-linear approaches for the controllability of networks are still restricted to single networks while it has been recently found that the multiplexity of networks can have profound effects on the dynamical processes taking place on them^39^,^40^,^41^,^42^,^43^,^44^. For example, percolation processes that usually present continuous phase transitions on single networks can become discontinuous on such structures^39^,^40^,^41^,^42^,^43^ and are characterized by large avalanches of disruption events.

Here, we consider the elegant framework of structural controllability^12^ and investigate how the multilayer structure of networks can affect their controllability. We focus on multiplex networks, which are multilayer networks in which the same set of nodes are connected by different types of interactions. Multiplex network controllability is studied under the assumption that input nodes are the same in all network layers, thus mimicking the situation in which input nodes can send different signals in the different layers of the multiplex but the position of the external signals in the layers is correlated.

We show that controlling the dynamics of multiplex networks is more costly than controlling single layers taken in isolation. Moreover, the controllability of multiplex networks displays unexpected new phenomena. In fact these networks can become extremely sensible to damage in conjunction with a discontinuous phase transition characterized by a jump in the number of input points (driver nodes). A careful investigation of this phase transition reveals that this is a hybrid phase transition with a square root singularity, therefore in the same universality class of the emergence of the mutually connected component in multiplex networks^1^,^39^,^41^. The number of driver nodes in the multiplex network is in general higher than the number of driver nodes in the single layers taken in isolation. Nevertheless the degree correlations between low-degree nodes in the different layers can affect the controllability of the multiplex network and modulate the number of its driver nodes. Moreover, a fully controllable configuration can be stable in a multilayer network even if it is not stable in the isolated networks that form the multilayer structure.

## Results

**Figure 1:**
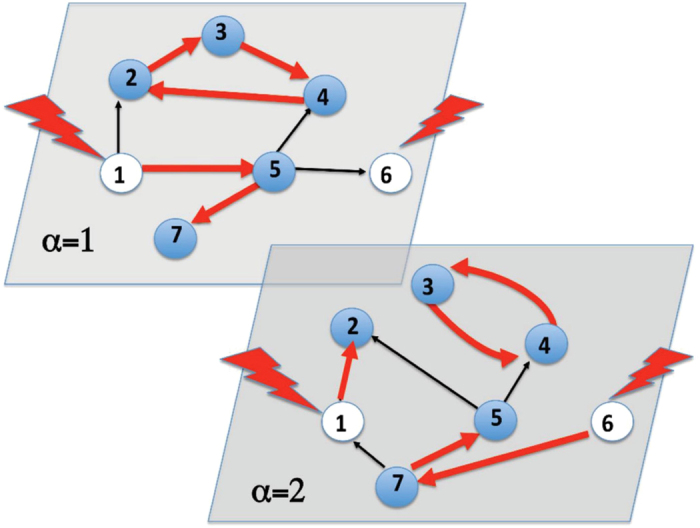
Control of a multiplex network. The controllability of a duplex network (multiplex with *M* = 2 layers) can be mapped to a Maximum Matching Problem in which the unmatched nodes (indicated with a white circle) are the driver nodes of the duplex network. Here we have indicated with red thick links the matched links and by black thin links the unmatched links.

We consider multiplex networks^1^ in which every node 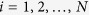 has a replica node (*i*, *α*) in each layer *α* and every layer is formed by a directed network between the corresponding replica nodes. We assume that each replica node (*i*, *α*) is characterized by a different dynamical variable 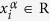 and that each layer is characterized by a possibly different dynamical process. We consider for simplicity a duplex, i.e. a multiplex formed by two layers {*A*, *B*} where each layer 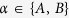 is a directed network. The state of the network at time *t* is governed by a linear dynamical system


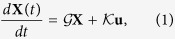


in which the 2*N*-dimensional vector **X**(*t*) describes the dynamical state of each replica node, i.e. 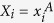 and 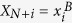 for 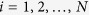. The matrix 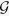 is a 2*N* × 2*N* (asymmetric) matrix and 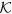 is a 2*N* × *P* matrix. They have the following block structure


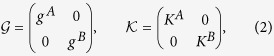


in which *g*^*α*^ are the *N* × *N* matrices describing the directed weighted interactions within the layers and *K*^*α*^ are the *N* × *P*^*α*^ matrices describing the coupling between the nodes of each layer *α* and *P*^*α*^ ≤ *N* external signals. The latter are represented by a vector **u**(*t*) of elements *u*_*γ*_ and 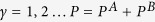. Here we consider the concept of structural controllability^11^,^12^ that guarantees the controllability of a networks for any choice of the non-zeros entries of 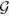 and 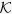, except for a variety of zero Lebesgue measure in the parameter space. Therefore each layer of the duplex networks can be structurally controlled by identifying a minimum number of *driver nodes*, that are controlled nodes which do not share input vertices. If different replicas of the same node can be independently controlled, then the controllability properties of the multiplex network factorize and each layer can be studied as if was taken in isolation^11^,^12^,^16^,^20^. Liu *et al.*^12^ showed that in a single layer the minimum set of driver nodes can be found by mapping the problem into a matching problem. In real multiplex networks however nodes are usually univocally defined and share common properties across different layers, therefore we make the assumption that *each node of the duplex network is either a driver node in each layer or it is not a driver node in any layer*. The problem of finding the driver nodes of the duplex network can be thus mapped into a maximum matching problem in which every node has at most one matched incoming link and at most one matched outgoing link, with the constraint that two replica nodes either have no matched incoming links on each layer or have one matched incoming link in each layer (see Fig. 1). This problem can be studied, using statistical mechanics techniques, such as the cavity method and the Belief Propagation (BP) algorithm. Following^12^,^16^, we consider the variables 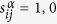 indicating respectively if the directed link from node 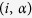 to node 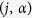 in layer 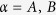 is matched or not. In order to have a matching in each layer of the duplex the following constraints have always to be satisfied


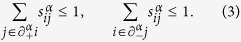


where 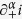 is the set of replica nodes 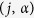 in layer *α* that are reached by directed links from 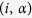 and 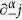 is the set of replica nodes 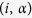 in layer *α* that point to 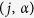. In addition, we impose that the driver nodes in the two layers (the unmatched nodes) are replica nodes, i.e.


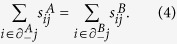


In this formalism, computing the maximum matching corresponds to minimize an energy function 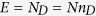 where *N*_*D*_ is the number of unmatched replica nodes associated to each matching. The energy *E* for a given matching, can be expressed in terms of the variables *s*_*ij*_ as


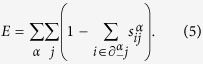


In order to study this novel statistical mechanics problem, we derived the BP equations^33^,^35^,^36^ (see Methods and Supplementary Material) valid in the locally tree-like approximation, as described for the case of a single network problem in^12^,^16^,^31^,^32^,^34^.

## Discussion

The controllability of multiplex networks displays a rich phenomenology, coming from the interplay between the dynamical and the structural properties of multiplex networks. Here we characterize the controllability of multiplex networks with different degree distribution and with tunable level of structural correlations.

### Phase transition in Poisson duplex networks

**Figure 2:**
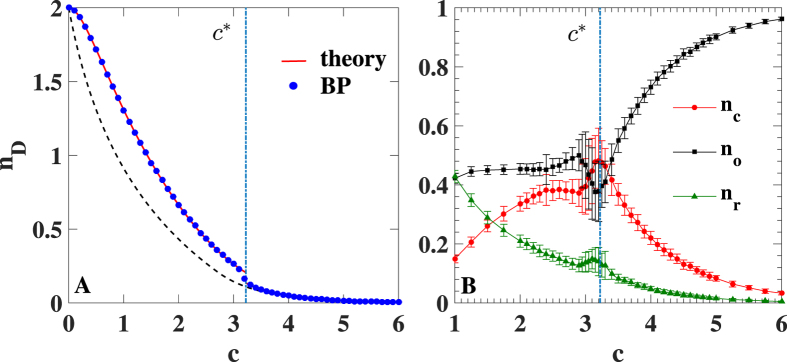
Controllability of Poisson duplex networks with average degrees 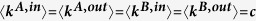. In panel (**A**) the fraction *n*_*D*_ of driver nodes in a Poisson duplex network with 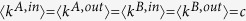, plotted as a function of the average degree *c*. The points indicate the average BP results obtained over 5 single realizations of the Poisson duplex networks with average degree *c* and *N* = 10^4^, the solid line is the theoretical expectation (the error bar, indicating the interval of one standard deviation from the mean, is always smaller or comparable to marker size). The dashed line represents twice the density of driver nodes for a single Poisson network with the same average degree. In panel (**B**) the densities *n*_*c*_, *n*_*r*_ and *n*_*o*_ respectively of critical redundant and ordinary nodes are shown as functions of *c* for the same type of duplex networks with *N* = 10^3^, where each point is the average over 100 different instances. In both panels the dot-dashed vertical line indicates the phase transition average degree 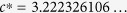.

We consider duplex networks in which the two layers are realizations of uncorrelated directed random graphs characterized by Poisson distributions for in-degree and out-degree with same average value *c*, i.e. 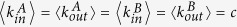. In Fig. 2A we report the average rescaled number of driver nodes *n*_*D*_ as function of the average degree *c* computed from the solutions of Eq. (8) on single instances and from the graph ensemble analysis. A comparison with two independent layers with the same topological properties shows that the controllability of a duplex network is in general more demanding in terms of number of driver nodes than the controllability of independent single layers, in particular for low average degrees. In addition, a discontinuity in the number of driver nodes at 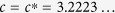 marks a change in the controllability properties of duplex networks that is not observed in uncoupled networks. This is due to a structural change in the solution of the matching problem, in which a finite density of zero valued cavity fields emerges. A careful investigation (see Supplementary Material) reveals that this is a hybrid phase transition with a square root singularity, therefore in the same universality class of the emergence of the mutually connected component in multiplex networks^1^,^39^,^41^.

In correspondence to this phase transition the network responds non trivially to perturbations. This is observed by performing a numerical calculation of the robustness of the networks. Following^12^ we classify the nodes into three categories: *critical nodes*, *redundant nodes* and *ordinary nodes*. When a critical node is removed from the (multiplex) network, controllability is sustained at the cost of increasing the number of driver nodes. If the number of driver nodes decreases or is unchanged, the removed nodes are classified as redundant and ordinary respectively. Figure 2B shows that the fraction of critical nodes reaches a maximum at the transition, revealing an increased fragility of the duplex network to random damage with respect to single layers. While an abrupt change in the number of driver nodes can result from a small change in the network topology, it is important to stress that the non-monotonic behavior of these quantities around the critical average degree value could be interpreted as a precursor of the discontinuity.

**Figure 3:**
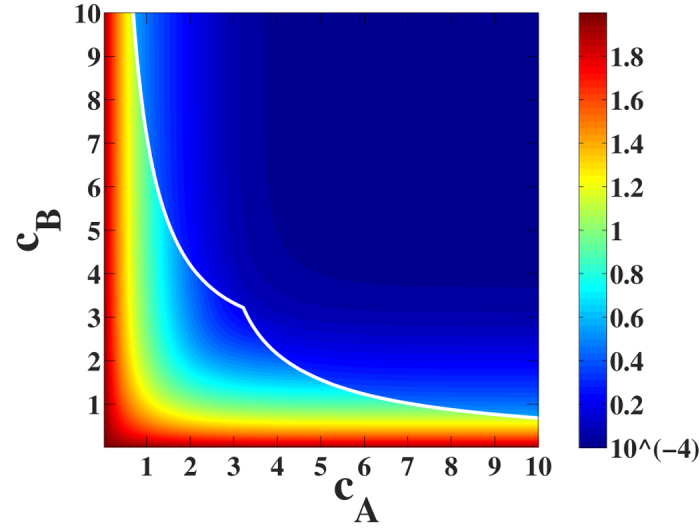
Phase diagram of the controllability for a Poisson duplex networks with average degrees 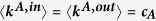 and 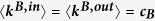. The color code indicates the density of driver nodes *n*_*D*_ = *E*/*N* in the multiplex network.

In a duplex network formed by directed Poisson random graphs with different average degree in the two layers (i.e. 
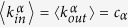
) a similar discontinuous phase transition is observed (see Fig. 3).

### Effect of degree correlations on the controllability of duplex networks

**Figure 4:**
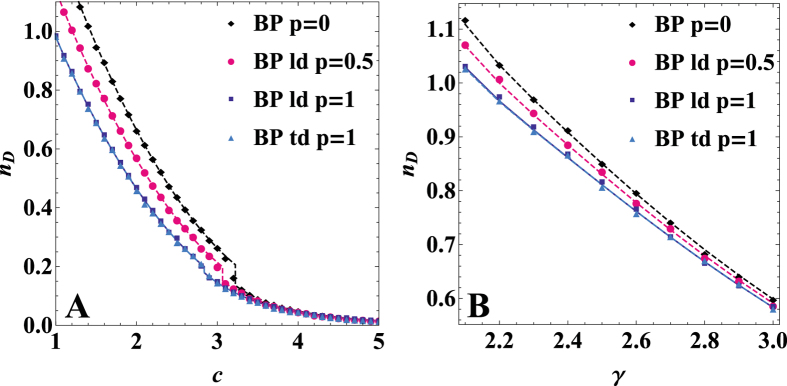
The effect of the degree correlation between replica nodes in different layers on the controllability of multiplex networks. Correlations between the low in-degrees (ld) and correlations between any in-degree node (td), parametrized by *p*, affect the fraction of driver nodes in the network *n*_*D*_, both in the case of Poisson networks with the same in and out average degree *c* across the two layers (Panel **A**) and in the case of scale-free networks with the same in and out degree distribution across the layers, given by 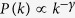 and minimum in/out degree 1 (Panel **B**). When *p* = 0 there is no degree correlation between replica nodes in different layers. The BP data are shown for networks with *N* = 10^4^, and are averaged 5 times for panel A and 20 times for panel **B**.

We consider a model of duplex network in which the replica nodes of the directed random graphs in the two layers have correlated degrees. In particular, we consider a case in which only the low in-degree nodes (nodes with in-degree equal to 0, 1, 2) are correlated (replica nodes in different layers have same degree with probability *p*) and a case in which the in-degrees of the replica nodes are equal with probability *p* independently of their value (see Supplementary Material for details). The controllability of the network is affected by these correlations as shown in Fig. 4. In fact, the number of driver nodes *n*_*D*_ decreases as the level of correlation increases. In duplex networks with Poisson degree distribution, low-degree correlations modify both the position of the hybrid transition and the size of the discontinuity. Once the replica nodes with low in-degree are correlated, a further correlation of the remaining replica nodes does not substantially change the number of driver nodes. This result confirms that structural controllability of random tree-like networks is essentially determined by the control of low degree nodes^16^.

### Stability of the fully controllable solution

A fully controllable solution, in which a single driver node is necessary to control the whole duplex network, exists if the minimum in-degree and the minimum out-degree are both greater than 1 in both layers. This solution of the cavity equations gives the correct solution to the maximum matching problem describing the controllability of multilayer networks only if no instabilities take place. The stability conditions are then found by imposing that the Jacobian of the systems of equations derived by the cavity method has all its eigenvalues *λ*_*i*_ of modulus less than one, i.e. 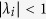. In random duplex networks with the same degree distribution in the two layers, the fully controllable solution is stable (see Supplementary Material for the details of the derivation) if and only if


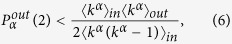


for *α* = *A*, *B*. On single random networks it was instead recently found^16^ that the fully controllable configuration is only stable for


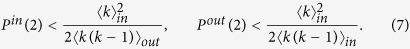


**Figure 5:**
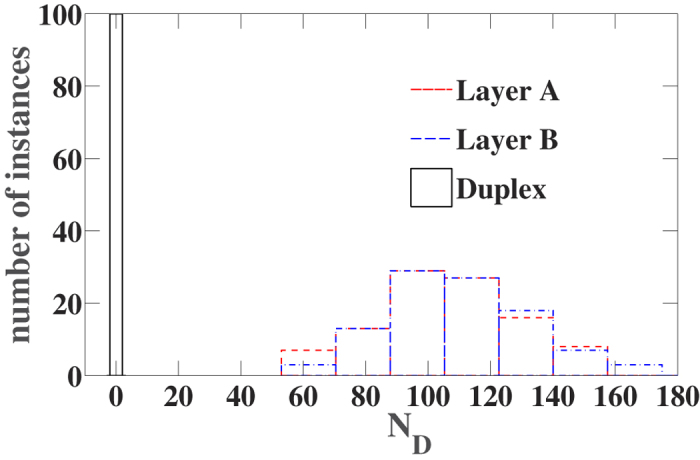
The fully controllable solution can be stable for the multiplex network also if it is not stable for the single layers taken in isolation. Histogram of the number of networks that out of 100 realizations have *N*_*D*_ driver nodes. The results obtained for the control of a duplex networks and its two layers are compared. The duplex networks are formed by two scale-free networks with *N* = 10^4^ and 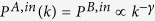 for *k* > 2 and 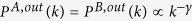 for *k* > 2, with *γ* = 2.3, the networks have minimum in-degree equal to 2 and minimum out-degree equal to 3 and 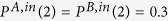.

This implies that for multiplex networks with asymmetric in-degree and out-degree distributions it might occur that the fully controllable solution is stable in the multiplex network but unstable in the single networks taken in isolation (see Fig. 5 for the characterization of the controllability of a similar type of multiplex networks). Therefore a multiplex structure can help to stabilize the fully controllable solution.

In conclusion, within the framework of structural controllability, we have considered the controllability properties of multiplex networks in which the nodes are either driver nodes in all the layers or they are not driver nodes in any layer. Our results show that controlling multiplex networks is more demanding, in terms of number of driver nodes, than controlling networks composed of a single layer. In random duplex networks with Poisson degree distribution, it is possible to observe a hybrid phase transition with a discontinuity in the number of driver nodes as a function of the average degree, that is phenomenologically similar to the emergence of mutually connected components. Close to this phase transition the duplex network exhibits an increased fragility to random damage. The existence of correlations between the degrees of replica nodes in different layers, in particular between low-degree nodes, has the effect of reducing the number of driver nodes necessary to control duplex networks. Finally, multiplex structure of networks can stabilize the fully controllable solution also if this solution is not stable in the single layers that form the multiplex network.

## Methods

### The BP equations

The BP equations of this problem are derived using the cavity method^33^,^35^,^36^ as described for the case of a single network problem in^12^,^16^,^31^,^32^,^34^. The same approximation methods can be applied here, as long as the structure of the interconnected layers is locally tree-like both within the layers and across them. Under the decorrelation (replica-symmetric) assumption, the cavity fields (or messages) 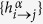 and 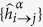, defined on the directed links between neighboring nodes 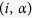 and 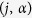 in the same layer *α* = *A*, *B* satisfy the zero-temperature limit of the BP equations, also known as Max-Sum equations,


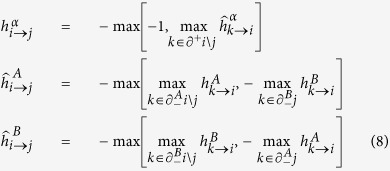


in which the fields are defined to take values in the discrete set 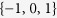 and here and in the following we use the convention that the maximum over a null set is equal to −1 (see Supplementary Material for details). In terms of these fields, the energy *E* in Eq. (5) becomes


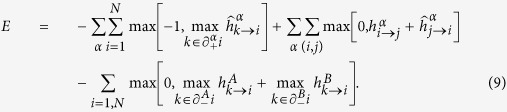


### BP equations over ensemble of networks

Let us consider the case of uncorrelated duplex networks in which the degree of the same node in different layers are uncorrelated and there is no overlap of the links. In each layer *α* = *A*, *B* we consider a maximally random network with in-degree distribution 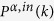 and out-degree distribution 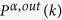. At the ensemble level, each link of (the infinitely large) random network forming layer *α* has the same statistical properties, that we describe through distributions 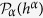 and 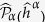 of cavity fields that are defined on the support of Eq. 8, i.e.


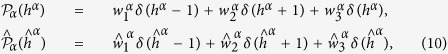


where *α* = *A*, *B* and where the probabilities 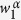, 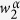, 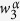 are normalized 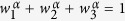 as well as the probabilities 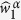, 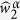, 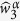 that satisfy the equation 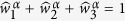. The cavity method at the network ensemble level is also known as density evolution method^36^.

It is useful to introduce the generating functions 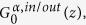 and 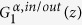 of the multiplex network as 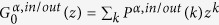, 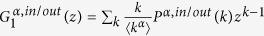, with *α* = *A*, *B*. In this way, we can derive recursive equations for the probabilities 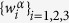 and 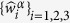, that are the analogous of the BP equations for an ensemble of uncorrelated duplex networks


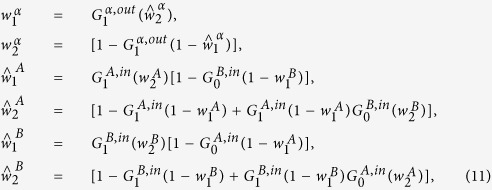


with 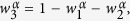 and 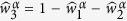. The energy *E* and the entropy density *s* of the matching problem can be also expressed in terms of the 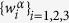 and 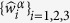 (see Supplementary Material for details).

### Hybrid transition for Poisson duplex network

Here we consider the case of two Poisson networks with the same in/out average degree. In other words, we consider the situation in which 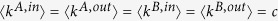. We notice that the BP equations can be rewritten to form a closed subsystem of equations for 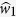 and 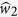 (see Supplementary Material for details),


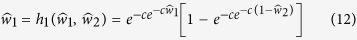



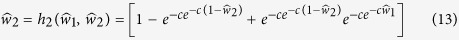


from the solution of which the remaining quantities can be determined.

The value 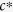 of the average degree *c* at which the discontinuity in the number of driver nodes *n*_*D*_ observed is found by imposing that Eqs. (12, 13) are satisfied together with the condition


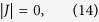


with *J* indicating the Jacobian of the system of equations (12, 13). Imposing that Eqs. (12, 13) and condition (14) are simultaneously satisfied, the solution 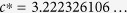 is found. For 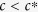 we observe that 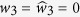. At 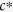 we observe a discontinuity in both *w*_3_ and 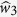, but for 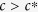 the functions 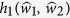 and 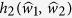 are analytic, and analyzing Eqs. (12) and (13) we obtain the behaviour of the order parameters *w*_3_ and 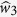 for 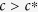



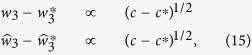


showing that the transition is hybrid.

## Additional Information

**How to cite this article**: Menichetti, G. *et al.* Control of Multilayer Networks. *Sci. Rep.* **6**, 20706; doi: 10.1038/srep20706 (2016).

## Supplementary Material

Supplementary Information
