# Supplementary material for: Control of Multilayer Networks
Source: Sci Rep. 2016 Feb 12;6:20706. doi: 10.1038/srep20706 (PMC4751610; doi:10.1038/srep20706)
Supplement: Supplementary Information [file srep20706-s1.pdf]

Supplemental Material "Control of Multilayer Networks"

Giulia Menichetti,[1] Luca Dall'Asta,[2,3] and Ginestra Bianconi[4]

[1]*Department of Physics and Astronomy and INFN Sez. Bologna, Bologna University, Viale B. Pichat 6/2 40127 Bologna, Italy*
[2]*Department of Applied Science and Technology DISAT, Politecnico di Torino, Corso Duca degli Abruzzi 24, 10129 Torino, Italy*
[3]*Collegio Carlo Alberto, Via Real Collegio 30, 10024 Moncalieri, Italy*
[4]*School of Mathematical Sciences, Queen Mary University of London, London E1 4NS, United Kingdom*

Contents

I. Introduction 1

II. The structural controllability of a multiplex network 1

III. The Maximum Matching Problem for the Controllability of Duplex Networks 2
A. Mapping duplex controllability into a constrained Maximum Matching Problem 2
B. Derivation of the BP equations at finite inverse temperature β 3
C. BP Equations for $\beta \to \infty$ 5

IV. Controllability of uncorrelated multiplex networks with given in-degree and out-degree distribution 5
A. Cavity equations for an uncorrelated multiplex network ensemble 5
B. Stability condition 6
C. Entropy 8
D. Phase transition in the controllability of Poisson duplex networks. 10
E. Controllability of scale-free duplex networks 13

V. Effect of degree correlations on controllability of multiplex networks 15

References 18

I. INTRODUCTION

This Supplemental Material is structured as follows.
In Sec. II we define the problem of structural controllability of multiplex networks, focusing on the case of a duplex network. Moreover we define the driver nodes, as the set of nodes that, if stimulated by an external signal, can drive the dynamical state of the network to any desired configuration.
In Sec. III we map the problem of structural controllability of a duplex network to a Maximum Matching Problem, and we derive the Belief Propagation (BP) equations determining the driver nodes, and their zero-temperature limit known as Max-Sum equations.
In Sec IV we consider the controllability of uncorrelated duplex networks, characterizing the BP equations valid for this problem, the stability conditions for the solutions of the BP equations, and the entropy of the solutions. Moreover, we consider duplex networks formed by two Poisson layers and we characterize their hybrid phase transition. Finally we consider the controllability of duplex networks formed by layers with power law in-degree and out-degree distributions.
In Sec. V we consider ensembles of duplex networks in which the in-degrees of replica nodes are correlated and we derive the BP equations assuming either that only the low in-degrees of replica nodes are correlated or that all the in-degrees of replica nodes are correlated.

II. THE STRUCTURAL CONTROLLABILITY OF A MULTIPLEX NETWORK

We consider a multiplex network in which every node $i = 1, 2, \ldots, N$ has a replica node in each layer and every layer is formed by a directed networks between the corresponding replica nodes [1]. We assume that each replica node can

have a different dynamical state and can send different signals in the different networks (each layers is characterized by a different dynamical process). In this case the controllability of the multiplex network can be treated by control theory methods used for the single layers taken in isolation [2–5]. Nevertheless here we will consider an additional constraint on the number of driver nodes. In fact we impose that corresponding replica nodes are either driver nodes in all layers or they are not driver nodes in any layer.

We consider for simplicity a duplex, i.e a multiplex formed by two layers where each layer is formed by a directed network. We call the two layers layer $\alpha = A, B$. We consider a linear dynamical system determining the network dynamics

$$\frac{d\mathbf{X}(t)}{dt} = \mathcal{G}\mathbf{X}(t) + \mathcal{K}\mathbf{u}(t), \tag{1}$$

in which the vector $\mathbf{X}(t)$ describes the dynamical state of each replica node in the duplex, and has $2N$ elements. The first set of N elements represents the dynamical state x_i^A of node i in layer A (i.e. $X_i = x_i^A$ for $i = 1, 2 \ldots, N$), while the elements X_{N+i} represent the dynamical state of the node i in layer B, and are given by $X_{N+i} = x_i^B$ for $i = 1, 2, \ldots, N$. The matrix $\mathcal{G}$ is a $2N \times 2N$ (asymmetric) matrix and the matrix $\mathcal{K}$ is a $2N \times M$ matrix. The matrices $\mathcal{G}$ and $\mathcal{K}$ have the following block structure

$$\mathcal{G} = \begin{pmatrix} g^A & 0 \\ 0 & g^B \end{pmatrix}, \quad \mathcal{K} = \begin{pmatrix} K^A & 0 \\ 0 & K^B \end{pmatrix}, \tag{2}$$

where g^α with $\alpha = A, B$ are the $N \times N$ matrices describing the directed weighted interactions within each of the networks in the two layers and K^α are the $N \times M^\alpha$ matrices describing the interaction between the nodes of the network α and the $M^\alpha \leq N$ external signals for layer α. The external signals are indicated by the vector $\mathbf{u}(t)$ of elements u_γ and $\gamma = 1, 2 \ldots M = M^A + M^B$.

Given block structure of both matrix $\mathcal{G}$ and $\mathcal{K}$ described in Eq. (2), the problem of duplex network controllability defined by Eq. (1), can be exactly recast into the problem of controllability of the single layers that form the duplex network.

Here we adopt the framework of *structural controllability* [3] aimed at characterizing if a given duplex network is controllable when the non-zero matrix elements of $\mathcal{G}$ and $\mathcal{K}$ given by Eq. (2) are free parameters. A duplex networks in which the linear dynamics described by the Eqs. (1) and (2) take place, is structurally controllable if both layers $\alpha = A, B$ are structurally controllable.

Each layer α is structurally controllable if for any choice of the free parameters in g^α and K^α, except for a variety of zero Lebesgue measure in the parameter space, the Kalman's condition is fulfilled [3]. Since structural controllability only distinguishes between zero and non-zero entries of the matrices g^α and K^α, a given directed network in layer α is structurally controllable if it is possible to determine the input nodes (i.e. the position of the non-zero entries of the matrix K^α) in a way to control the dynamics described by any realization of the matrix g^α with the same non-zero elements, except for atypical realizations of zero measure. In practice, a single network can be structurally controlled by identifying a minimum number of *driver nodes*, that are controlled nodes which do not share input vertices in both layers. In their seminal paper [4], Liu and coworkers showed that on single networks this control theoretic problem can be reduced to a well-known optimization problem: their Minimum Input Theorem states that the minimum set of driver nodes that guarantees the full structural controllability of a network is the set of unmatched nodes in a maximum matching of the same directed network. Their result for a single network remains valid for the duplex network described by Eqs. $(1) - (2)$. Therefore the structural controllability of duplex networks, in the absence of further constraints can be mapped to a Maximum Matching problem defined on the single layers of the duplex networks. Here nevertheless, we consider a further constraint to be imposed on the driver nodes, which enforce a new type of dependence between the layers of the duplex. In particular we impose that the replica nodes (i, α) with $\alpha = A, B$ and a given index i, are either both driver nodes or neither is a driver node. This implies that these two replica nodes are either both linked to independent and external signals or none of them is connected to external signals.

III. THE MAXIMUM MATCHING PROBLEM FOR THE CONTROLLABILITY OF DUPLEX NETWORKS

A. Mapping duplex controllability into a constrained Maximum Matching Problem

In order to build an algorithm able to find the driver nodes of a duplex network we consider the variables $s_{ij}^\alpha = 1, 0$ indicating respectively if the directed link from node (i, α) to node (j, α) in layer $\alpha = A, B$ is matched or not. In the

two layers of the duplex network we want to have a matching, i.e. the following constraints must always be satisfied for $\alpha = A, B$,

$$\sum_{j\in\partial_+^\alpha i} s_{ij}^\alpha \ \leq \ 1, \tag{3a}$$

$$\sum_{j\in\partial_-^\alpha i} s_{ji}^\alpha \ \leq \ 1, \tag{3b}$$

where here and in the following we indicate with ∂_+^α the set of nodes j that are pointed by node i in layer α and with $\partial_-^\alpha i$ the set of nodes j pointing to node i in layer α. In addition we impose that the driver nodes in the two networks are replica nodes, i.e. in the matching problem either two replica nodes are both matched or both unmatched. Therefore the variable s_{ij}^α satisfy the following additional constraints

$$\sum_{i\in\partial_-^A j} s_{ji}^A = \sum_{i\in\partial_-^B j} s_{ji}^B. \tag{4}$$

Finally we need to minimize the number of driver nodes in the multiplex network. Therefore we minimize the energy E of the problem given by

$$E \ = \ \sum_\alpha \sum_j \left(1 - \sum_{i\in\partial_-^\alpha j} s_{ij}^\alpha\right) = \sum_\alpha \sum_i E_i^\alpha, \tag{5}$$

with

$$E_i^\alpha = 1 - \sum_{j\in\partial_+^\alpha i} s_{ij}^\alpha. \tag{6}$$

The energy E is given by the number N_D of driver replica nodes in the duplex network by

$$E = N_D = N n_D. \tag{7}$$

B. Derivation of the BP equations at finite inverse temperature β

We consider here the Maximum Matching Problem defined in Sec III A. The goal is to find the configuration of the variables $\{s_{ij}^\alpha\}$ associated to every directed edge $i \to j$ in layer α, such that the energy E given by the number of driver replica nodes in the duplex network is minimized provided that the conditions given by Eqs. (3), (4) are satisfied. Introducing as an auxiliary variable the "inverse temperature" β we cast this problem into a statistical mechanics problem where our first aim is finding the distribution $P(\{s_{ij}\})$, parametrized by the *inverse temperature* β, and given by

$$P(\{s_{ij}\}) \ = \ \frac{e^{-\beta E}}{Z}\prod_{i=1}^N\left\{\prod_\alpha\left[\theta\left(1-\sum_{j\in\partial_+ i} s_{ij}^\alpha\right)\theta\left(1-\sum_{j\in\partial_- i} s_{ji}^\alpha\right)\right]\delta\left(\sum_{i\in\partial_-^A j} s_{ij}^A, \sum_{i\in\partial_-^B j} s_{ij}^B\right)\right\}, \tag{8}$$

where $\theta(x) = 1$ for $x \geq 0$ and $\theta(x) = 0$ for $x < 0$, $\delta(x)$ is the Kronecker delta, and where Z is the normalization constant, that corresponds to the partition function of the statistical mechanics problem. Subsequently, we plan to perform the limit $\beta \to \infty$ in order to characterize the optimal (i.e. the maximum-sized) matching in the network satisfying Eqs. (3), (4). The free-energy of the problem $F(\beta)$ is defined as

$$\beta F(\beta) = -\ln Z, \tag{9}$$

and the energy E is therefore given by

$$E = \frac{\partial[\beta F(\beta)]}{\partial\beta}. \tag{10}$$

The distribution $P(\{s_{ij}\})$ on a locally tree-like network can be (approximately) estimated by the cavity method in the replica symmetric assumption (i.e. by deriving Belief Propagation equations) [4–11]. In this respect, in each layer

α of the duplex network, we define two probability marginals on each directed link, one going in the same direction of the link $P^{\alpha}_{i\to j}(s_{ij})$ and one in the opposite direction $\hat{P}^{\alpha}_{i\to j}(s_{ji})$. The BP equations for these quantities are

$$P^{\alpha}_{i\to j}(s_{ij}) = \frac{1}{\mathcal{D}^{\alpha}_{i\to j}} \sum_{\{s^{\alpha}_{ik}\}|k\in\partial^{\alpha}_{+}i\setminus j} \left\{\theta\left(1-\sum_{k\in\partial^{\alpha}_{+}i} s^{\alpha}_{ik}\right)\exp\left[-\beta\left(1-\sum_{k\in\partial^{\alpha}_{+}i} s^{\alpha}_{ik}\right)\right]\prod_{k\in\partial^{\alpha}_{+}i\setminus j}\hat{P}^{\alpha}_{k\to i}(s^{\alpha}_{ik})\right\}, \tag{11a}$$

$$\hat{P}^{A}_{i\to j}(s^{A}_{ji}) = \frac{1}{\hat{\mathcal{D}}^{A}_{i\to j}} \sum_{\{s^{A}_{ki}\}\setminus s^{A}_{ji}, k\in\partial^{A}_{-}i} \left\{\theta\left(1-\sum_{k\in\partial^{A}_{-}i} s^{A}_{ki}\right)\sum_{\{s^{B}_{ki}\}|k\in\partial^{B}_{-}i}\left[\theta\left(1-\sum_{k\in\partial^{B}_{-}i} s^{B}_{ki}\right)\delta\left(\sum_{i\in\partial^{A}_{-}j} s^{A}_{ij}, \sum_{i\in\partial^{B}_{-}j} s^{B}_{ij}\right)\right.\right.$$
$$\left.\left.\times\prod_{k\in\partial^{A}_{-}i\setminus j} P^{A}_{k\to i}(s^{A}_{ki})\prod_{k\in\partial^{B}_{-}i} P^{B}_{k\to i}(s^{B}_{ki})\right]\right\}, \tag{11b}$$

$$\hat{P}^{B}_{i\to j}(s^{B}_{ji}) = \frac{1}{\hat{\mathcal{D}}^{B}_{i\to j}} \sum_{\{s^{B}_{ki}\}|k\in\partial^{B}_{-}\setminus j} \left\{\theta\left(1-\sum_{k\in\partial^{B}_{-}i} s^{B}_{ki}\right)\sum_{\{s^{A}_{ki}\}|k\in\partial^{A}_{-}i}\left[\theta\left(1-\sum_{k\in\partial^{A}_{-}i} s^{A}_{ki}\right)\delta\left(\sum_{i\in\partial^{A}_{-}j} s^{A}_{ij}, \sum_{i\in\partial^{B}_{-}j} s^{B}_{ij}\right)\right.\right.$$
$$\left.\left.\times\prod_{k\in\partial^{B}_{-}i\setminus j} P^{B}_{k\to i}(s^{B}_{ki})\prod_{k\in\partial^{A}_{-}i} P^{A}_{k\to i}(s^{A}_{ki})\right]\right\} \tag{11c}$$

where $\mathcal{D}^{\alpha}_{i\to j}$ and $\hat{\mathcal{D}}^{\alpha}_{i\to j}$ are normalization constants. The probability marginals $\{P^{\alpha}_{i\to j}(s^{\alpha}_{ij}),\ \hat{P}^{\alpha}_{i\to j}(s^{\alpha}_{ji})\}$ can be parametrized by the cavity fields $h^{\alpha}_{i\to j}$ and $\hat{h}^{\alpha}_{i\to j}$ defined by

$$P^{\alpha}_{i\to j}(s^{\alpha}_{ij}) = \frac{\exp\left[\beta h^{\alpha}_{i\to j}s^{\alpha}_{ij}\right]}{1+\exp\left[\beta h^{\alpha}_{i\to j}\right]} \qquad \hat{P}^{\alpha}_{i\to j}(s^{\alpha}_{ji}) = \frac{\exp\left[\beta \hat{h}^{\alpha}_{i\to j}s^{\alpha}_{ji}\right]}{1+\exp\left[\beta \hat{h}^{\alpha}_{i\to j}\right]}. \tag{12}$$

In terms of the cavity fields (or messages), Eqs. (11) reduce to the following set of finite temperature BP equations,

$$h^{\alpha}_{i\to j} = -\frac{1}{\beta}\log\left(e^{-\beta}+\sum_{k\in\partial^{\alpha}_{+}i\setminus j} e^{\beta\hat{h}^{\alpha}_{k\to i}}\right), \tag{13a}$$

$$\hat{h}^{A}_{i\to j} = -\frac{1}{\beta}\log\left(\frac{1}{\sum_{k\in\partial^{B}_{-}i} e^{\beta h^{B}_{k\to i}}}+\sum_{k\in\partial^{A}_{-}i\setminus j} e^{\beta h^{A}_{k\to i}}\right), \tag{13b}$$

$$\hat{h}^{B}_{i\to j} = -\frac{1}{\beta}\log\left(\frac{1}{\sum_{k\in\partial^{A}_{-}i} e^{\beta h^{A}_{k\to j}}}+\sum_{k\in\partial^{B}_{-}i\setminus j} e^{\beta h^{B}_{k\to i}}\right), \tag{13c}$$

The free energy F and the energy $E = \frac{\partial \beta F}{\partial \beta}$ of the model are given respectively by

$$-\beta F = \sum_{\alpha}\sum_{i=1}^{N}\left[\ln\left(e^{-\beta}+\sum_{k\in\partial^{\alpha}_{+}i} e^{\beta\hat{h}^{\alpha}_{k\to i}}\right)\right]+\sum_{i=1,N}\ln\left(1+\sum_{k\in\partial^{A}_{-}i} e^{\beta h^{A}_{k\to i}}\sum_{k'\in\partial^{B}_{-}i} e^{\beta h^{B}_{k'\to i}}\right)$$
$$-\sum_{\alpha}\sum_{<i,j>_{\alpha}}\ln\left(1+e^{\beta(h^{\alpha}_{i\to j}+\hat{h}^{\alpha}_{j\to i})}\right), \tag{14}$$

and by

$$E = \sum_{\alpha}\sum_{i=1}^{N}\left[\frac{e^{-\beta}-\sum_{k\in\partial^{\alpha}_{+}i}\hat{h}^{\alpha}_{k\to i}e^{\beta\hat{h}^{\alpha}_{k\to i}}}{1+\sum_{k\in\partial^{\alpha}_{+}i} e^{\beta\hat{h}^{\alpha}_{k\to i}}}\right]-\sum_{i=1}^{N}\frac{\sum_{k\in\partial^{A}_{-}i} h^{A}_{k\to i}e^{\beta h^{A}_{k\to i}}\sum_{k'\in\partial^{B}_{-}i} e^{\beta h^{B}_{k'\to i}}}{1+\sum_{k\in\partial^{A}_{-}i} e^{\beta h^{A}_{k\to i}}\sum_{k'\in\partial^{B}_{-}i} e^{\beta h^{B}_{k'\to i}}}$$
$$-\sum_{i}\frac{\sum_{k\in\partial^{A}_{-}i} e^{\beta h^{A}_{k\to i}}\sum_{k'\in\partial^{B}_{-}i} h^{B}_{k'\to i}e^{\beta h^{B}_{k'\to i}}}{1+\sum_{k\in\partial^{A}_{-}i} e^{\beta h^{A}_{k\to i}}\sum_{k'\in\partial^{B}_{-}i} e^{\beta h^{B}_{k'\to i}}}+\sum_{\alpha}\sum_{<i,j>_{\alpha}}\frac{(h^{\alpha}_{i\to j}+\hat{h}^{\alpha}_{j\to i})e^{\beta(h^{\alpha}_{i\to j}+\hat{h}^{\alpha}_{j\to i})}}{1+e^{\beta(h^{\alpha}_{i\to j}+\hat{h}^{\alpha}_{j\to i})}}. \tag{15}$$

C. BP Equations for $\beta \to \infty$

The BP equations in the limit $\beta \to \infty$ are derived from the Eqs. (13). In the limit $\beta \to \infty$ the solution is expressed in terms of the fields $h^{\alpha}_{i\to j}$ or $\hat{h}^{\alpha}_{i\to j}$ sent from a node (i,α) to the linked node (j,α) in layer $\alpha = A, B$. The cavity fields have a simple interpretation as messages between neighboring replica nodes [6]: $h^{\alpha}_{i\to j} = \hat{h}^{\alpha}_{i\to j} = 1$ means *"match me"*, $h^{\alpha}_{i\to j} = \hat{h}^{\alpha}_{i\to j} = -1$ means *"do not match me"*, and $h^{\alpha}_{i\to j} = \hat{h}^{\alpha}_{i\to j} = 0$ means *"do what you want"*. The zero-temperature BP (or Max-Sum) equations determining the values of these fields in the limit $\beta \to \infty$ are are given by

$$
\begin{aligned}
h^{\alpha}_{i\to j} &= -\max\left[-1, \max_{k\in\partial^{+}i\setminus j} \hat{h}^{\alpha}_{k\to i}\right] &\quad (16a)\\
\hat{h}^{A}_{i\to j} &= -\max\left[\max_{k\in\partial^{A}_{-}i\setminus j} h^{A}_{k\to i}, -\max_{k\in\partial^{B}_{-}j} h^{B}_{k\to i}\right] &\quad (16b)\\
\hat{h}^{B}_{i\to j} &= -\max\left[\max_{k\in\partial^{B}_{-}i\setminus j} h^{B}_{k\to i}, -\max_{k\in\partial^{A}_{-}j} h^{A}_{k\to i}\right] &\quad (16c)
\end{aligned}
$$

in which the fields are defined to take values in the discrete set $\{1, 0, -1\}$ and we defined the maximum over a null set equal to -1. It follows that for $k_i^{B,in} = 0$ we have $\hat{h}^{A}_{i\to j} = -1$ and for $k_i^{A,in} = 0$ we have $\hat{h}^{B}_{i\to j} = -1$.

The energy E can also be expressed in terms of these fields and is given by

$$
\begin{aligned}
E &= -\sum_{\alpha}\sum_{i=1}^{N} \max\left[-1, \max_{k\in\partial^{\alpha}_{+}i} \hat{h}^{\alpha}_{k\to i}\right] \\
&+\sum_{\alpha}\sum_{<i,j>} \max\left[0, h^{\alpha}_{i\to j} + \hat{h}^{\alpha}_{j\to i}\right] \\
&-\sum_{i=1,N} \max\left[0, \max_{k\in\partial^{A}_{-}i} h^{A}_{k\to i} + \max_{k\in\partial^{B}_{-}i} h^{B}_{k\to i}\right], \quad (17)
\end{aligned}
$$

where $< i, j >$ indicates pair of nodes that are nearest neighbors in the network and where we take the maximum over a null set equal to -1.

IV. CONTROLLABILITY OF UNCORRELATED MULTIPLEX NETWORKS WITH GIVEN IN-DEGREE AND OUT-DEGREE DISTRIBUTION

A. Cavity equations for an uncorrelated multiplex network ensemble

Let us consider the case of uncorrelated duplex networks in which the degree of the same node in different layers are uncorrelated and there is no overlap of the links. In each layer $\alpha = A, B$ we consider a maximally random network with in-degree distribution $P^{\alpha,in}(k)$ and out-degree distribution $P^{\alpha,out}(k)$. At the ensemble level, each link of (the infinitely large) random network forming layer α has the same statistical properties, that we describe through distributions $\mathcal{P}_{\alpha}(h^{\alpha})$ and $\hat{\mathcal{P}}_{\alpha}(\hat{h}^{\alpha})$ of cavity fields that are defined on the support of Eqs.16, i.e.

$$
\begin{aligned}
\mathcal{P}_{\alpha}(h^{\alpha}) &= w_1^{\alpha}\delta(h^{\alpha}-1) + w_2^{\alpha}\delta(h^{\alpha}+1) + w_3^{\alpha}\delta(h^{\alpha}), \\
\hat{\mathcal{P}}_{\alpha}(\hat{h}^{\alpha}) &= \hat{w}_1^{\alpha}\delta(\hat{h}^{\alpha}-1) + \hat{w}_2^{\alpha}\delta(\hat{h}^{\alpha}+1) + \hat{w}_3^{\alpha}\delta(\hat{h}^{\alpha}), \quad (18)
\end{aligned}
$$

where $\alpha = A, B$ and where the probabilities $w_1^{\alpha}, w_2^{\alpha}, w_3^{\alpha}$ are normalized $w_1^{\alpha} + w_2^{\alpha} + w_3^{\alpha} = 1$ as well as the probabilities $\hat{w}_1^{\alpha}, \hat{w}_2^{\alpha}, \hat{w}_3^{\alpha}$ that satisfy the equation $\hat{w}_1^{\alpha} + \hat{w}_2^{\alpha} + \hat{w}_3^{\alpha} = 1$. The cavity method at the network ensemble level is also known as density evolution method [8].

It is useful to introduce the generating functions $G_0^{\alpha,in/out}(z)$, and $G_1^{\alpha,in/out}(z)$ of the multiplex network as

$$\begin{aligned} G_0^{\alpha,in}(z) &= \sum_k P^{\alpha,in}(k) z^k, \\ G_1^{\alpha,in}(z) &= \sum_k \frac{k}{\langle k^\alpha \rangle} P^{\alpha,in}(k) z^{k-1}, \\ G_0^{\alpha,out}(z) &= \sum_k P^{\alpha,out}(k) z^k, \\ G_1^{\alpha,out}(z) &= \sum_k \frac{k}{\langle k^\alpha \rangle} P^{\alpha,out}(k) z^{k-1}, \end{aligned} \tag{19}$$

with $\alpha = A, B$. In this way, we can derive recursive equations for the probabilities $\{w_i^\alpha\}_{i=1,2,3}$ and $\{\hat{w}_i^\alpha\}_{i=1,2,3}$, that are the analogous of Eqs. 16 for an ensemble of uncorrelated duplex networks

$$\begin{aligned} w_1^\alpha &= G_1^{\alpha,out}(\hat{w}_2^\alpha), \\ w_2^\alpha &= \left[1 - G_1^{\alpha,out}(1 - \hat{w}_1^\alpha)\right], \\ w_3^\alpha &= 1 - w_1^\alpha - w_2^\alpha, \\ \hat{w}_3^\alpha &= 1 - \hat{w}_1^\alpha - \hat{w}_2^\alpha, \\ \hat{w}_1^A &= G_1^{A,in}(w_2^A)\left[1 - G_0^{B,in}(1 - w_1^B)\right], \\ \hat{w}_2^A &= \left[1 - G_1^{A,in}(1 - w_1^A) + G_1^{A,in}(1 - w_1^A) G_0^{B,in}\left(w_2^B\right)\right], \\ \hat{w}_1^B &= G_1^{B,in}(w_2^B)\left[1 - G_0^{A,in}(1 - w_1^A)\right], \\ \hat{w}_2^B &= \left[1 - G_1^{B,in}(1 - w_1^B) + G_1^{B,in}(1 - w_1^B) G_0^{A,in}\left(w_2^A\right)\right]. \end{aligned} \tag{20}$$

The energy E of the matching problem can be also expressed in terms of the $\{w_i^\alpha\}_{i=1,2,3}$ and $\{\hat{w}_i^\alpha\}_{i=1,2,3}$ giving

$$\begin{aligned} E &= \sum_\alpha \left\{G_0^{\alpha,out}\left(\hat{w}_2^\alpha\right) - \left[1 - G_0^{\alpha,out}(1 - \hat{w}_1^\alpha)\right]\right\} - \Big\{[1 - G_0^{A,in}(1 - w_1^A)][1 - G_0^{B,in}(w_2^B)] \\ &\quad + [1 - G_0^{B,in}(1 - w_1^B)][1 - G_0^{A,in}(w_2^A)]\Big\} + \sum_\alpha \langle k^\alpha \rangle_{in}\left[\hat{w}_1^\alpha(1 - w_2^\alpha) + w_1^\alpha(1 - \hat{w}_2^\alpha)\right]. \end{aligned} \tag{21}$$

B. Stability condition

The Eqs.20 might have multiple solutions. In order to evaluate the stability of these solutions, using a method already used in the context of single networks [5, 6] here we compute the Jacobian of the system of Eqs. (20) and impose that all its eigenvalues have modulus less than one. We avoid to consider w_3^α and $\hat{w}_3^\alpha$ because they influence only the number of null eigenvalues (4 eigenvalues upon 12). The 12×12 Jacobian matrix becomes 8×8 and it can be decomposed in four 4×4 blocks

$$J = \begin{pmatrix} H_{11} & H_{1,2} \\ H_{2,1} & H_{2,2} \end{pmatrix}.$$

with

$$H_{11} = \begin{pmatrix} 0 & 0 & 0 & G_2^{A,out}(\hat{w}_2^A) \\ 0 & 0 & G_2^{A,out}(1 - \hat{w}_1^A) & 0 \\ 0 & G_2^{A,in}(w_2^A)(1 - G_0^{B,in}(1 - w_1^B)) & 0 & 0 \\ G_2^{A,in}(1 - w_1^A)(1 - G_0^{B,in}(w_2^B)) & 0 & 0 & 0 \end{pmatrix}, \tag{22}$$

$$H_{2,1} = \begin{pmatrix} 0 & 0 & 0 & 0 \\ 0 & 0 & 0 & 0 \\ G_1^{B,in}(w_2^B) \langle k \rangle_{A,in} G_1^{A,in}(1 - w_1^A) & 0 & 0 & 0 \\ 0 & G_1^{B,in}(1 - w_1^B) \langle k \rangle_{A,in} G_1^{A,in}(w_2^A) & 0 & 0 \end{pmatrix}, \tag{23}$$

$$H_{2,2}=\begin{pmatrix} 0 & 0 & 0 & 0 \\ 0 & 0 & 0 & 0 \\ G_1^{A,in}(w_2^A)\left\langle k\right\rangle_{B,in} G_1^{B,in}(1-w_1^B) & 0 & 0 & 0 \\ 0 & G_1^{A,in}(1-w_1^A)\left\langle k\right\rangle_{B,in} G_1^{B,in}(w_2^B) & 0 & 0 \end{pmatrix}, \tag{24}$$

and

$$H_{2,2}=\begin{pmatrix} 0 & 0 & 0 & G_2^{B,out}(\hat{w}_2^B) \\ 0 & 0 & G_2^{B,out}(1-\hat{w}_1^B) & 0 \\ 0 & G_2^{B,in}(w_2^B)(1-G_0^{A,in}(1-w_1^A)) & 0 & 0 \\ G_2^{B,in}(1-w_1^B)(1-G_0^{A,in}(w_2^A)) & 0 & 0 & 0 \end{pmatrix}. \tag{25}$$

Here the generating functions $G_0^{\alpha,in/out}$ and $G_1^{\alpha,in/out}$ are given by Eqs. (19) and the generating functions $G_2^{\alpha,in}(x)$ and $G_2^{\alpha,out}(x)$ are defined as

$$\begin{aligned} G_2^{\alpha,in}(z) &= \sum_k \frac{k(k-1)}{\left\langle k^\alpha\right\rangle_{in}} P_\alpha^{in}(k) z^{k-2} \\ G_2^{\alpha,out}(z) &= \sum_k \frac{k(k-1)}{\left\langle k^\alpha\right\rangle_{out}} P_\alpha^{out}(k) z^{k-2}. \end{aligned} \tag{26}$$

Of particular interest is the characterization of the stability of the solution $w_1^\alpha = \hat{w}_1^\alpha = w_2^\alpha = \hat{w}_2^\alpha = 0$ and $w_3^\alpha = \hat{w}_3^\alpha = 1$, corresponding to the full controllability of the network, a configuration with $E = N_D = 0$. This solution emerges for $P_\alpha^{in}(1) = P_\alpha^{out}(1) = 0$ for $\alpha = A, B$. Therefore if the minimum in-degree and the minimum out-degree are both greater than one, the analysis at the ensemble level is consistent with the full controllability of the network. Nevertheless this solution might be not stable. By analyzing the Jacobian J for $w_1^\alpha = \hat{w}_1^\alpha = w_2^\alpha = \hat{w}_2^\alpha = 0$ and $w_3^\alpha = \hat{w}_3^\alpha = 1$, we can determine under which condition the full controllability solution is stable. The Jacobian matrix, in this case simplify significantly and is given by

$$J=\begin{pmatrix} 0 & 0 & 0 & \frac{2P_A^{out}(2)}{\left\langle k^A\right\rangle_{out}} & 0 & 0 & 0 & 0 \\ 0 & 0 & \frac{\left\langle k^A(k^A-1)\right\rangle_{out}}{\left\langle k^A\right\rangle_{out}} & 0 & 0 & 0 & 0 & 0 \\ 0 & 0 & 0 & 0 & 0 & 0 & 0 & 0 \\ \frac{\left\langle k^A(k^A-1)\right\rangle_{in}}{\left\langle k^A\right\rangle_{in}} & 0 & 0 & 0 & 0 & 0 & 0 & 0 \\ 0 & 0 & 0 & 0 & 0 & 0 & 0 & \frac{2P_B^{out}(2)}{\left\langle k^B\right\rangle_{out}} \\ 0 & 0 & 0 & 0 & 0 & 0 & \frac{\left\langle k^B(k^B-1)\right\rangle_{out}}{\left\langle k^B\right\rangle_{out}} & 0 \\ 0 & 0 & 0 & 0 & 0 & 0 & 0 & 0 \\ 0 & 0 & 0 & 0 & \frac{\left\langle k^B(k^B-1)\right\rangle_{in}}{\left\langle k^B\right\rangle_{in}} & 0 & 0 & 0 \end{pmatrix} \tag{27}$$

Four eigenvalues of J are zero, the other four have degenerate modulus, therefore the stability conditions are

$$\begin{aligned} 2\frac{\left\langle k^A(k^A-1)\right\rangle_{in}}{\left\langle k^A\right\rangle_{in}}\frac{P_A^{out}(2)}{\left\langle k^A\right\rangle_{out}} &< 1 \\ 2\frac{\left\langle k^B(k^B-1)\right\rangle_{in}}{\left\langle k^B\right\rangle_{in}}\frac{P_B^{out}(2)}{\left\langle k^B\right\rangle_{out}} &< 1. \end{aligned} \tag{28}$$

When $P_A^{in}(k) = P_A^{out}(k) = P_B^{in}(k) = P_B^{out}(k) = P(k)$ we have just one stability criterion solution and it reads

$$P(2) < \frac{\left\langle k\right\rangle^2}{2\left\langle k(k-1)\right\rangle} \tag{29}$$

We observe here that on the single layers $\alpha = A, B$ the full controllability solution, is instead only stable [5] for

$$\begin{aligned} 2\frac{\left\langle k^\alpha(k^\alpha-1)\right\rangle_{in}}{\left\langle k^\alpha\right\rangle_{in}}\frac{P_\alpha^{out}(2)}{\left\langle k^\alpha\right\rangle_{out}} &< 1 \\ 2\frac{\left\langle k^\alpha(k^\alpha-1)\right\rangle_{out}}{\left\langle k^\alpha\right\rangle_{out}}\frac{P_\alpha^{in}(2)}{\left\langle k^\alpha\right\rangle_{in}} &< 1. \end{aligned} \tag{30}$$

It follows that for duplex networks in which both layers have the same in-degree and out-degree distributions, i.e. $P^{\alpha,in}(k) = P^{\alpha,out}(k)$ the stability of the full controllability solution on single layers is the same as the stability on the duplex network. Nevertheless, for duplex networks formed by layers in which the in-degree distribution and the out-degree distribution are not the same there can be cases in which for the duplex network the fully controllable solution is stable while for the single layers it is not stable (See main text for discussion of this phenomenon and simulation results).

C. Entropy

In order to evaluate the number of maximum matchings, here we evaluate the entropy of the ground state solutions in the case of uncorrelated layers. The entropy density is given by $s_0 = S_0/N$ and can be computed by expanding the free energy at low temperatures $f(\beta \to \infty) = e_0 - s_0/\beta + \mathcal{O}(1/\beta^2)$. This involves the study of the evanescent parts of the cavity field. Therefore we assume that the field can be written as

$$\begin{array}{rll} h_\alpha = 1 + \frac{\ln \nu_\alpha}{\beta} & \text{for the peak around} & h = 1 \\ h_\alpha = -1 + \frac{\ln \mu_\alpha}{\beta} & \text{for the peak around} & h = -1 \\ h_\alpha = \frac{\ln \gamma_\alpha}{\beta} & \text{for the peak around} & h = 0 \end{array}$$

$$\begin{array}{rll} \hat{h}_\alpha = 1 + \frac{\ln \hat{\nu}_\alpha}{\beta} & \text{for the peak around} & h = 1 \\ \hat{h}_\alpha = -1 + \frac{\ln \hat{\mu}_\alpha}{\beta} & \text{for the peak around} & h = -1 \\ \hat{h}_\alpha = \frac{\ln \hat{\gamma}_\alpha}{\beta} & \text{for the peak around} & h = 0 \end{array}$$

From the BP equations, and the equations for $P(h_\alpha)$ and $P(\hat{h}_\alpha)$ we can obtain the relation between the probability distributions $\mathcal{A}_1^\alpha(\nu_\alpha), \mathcal{A}_2^\alpha(\mu_\alpha), \mathcal{A}_3^\alpha(\gamma_\alpha)$, and the distributions $\hat{\mathcal{A}}_1^\alpha(\hat{\nu}_\alpha), \hat{\mathcal{A}}_2^\alpha(\mu_\alpha), \hat{\mathcal{A}}_3^\alpha(\hat{\gamma}_\alpha)$, given by

$$\mathcal{A}_1^\alpha(\nu) = \sum_{k=0}^{\infty} \frac{(\hat{w}_2^\alpha)^k}{w_1^\alpha} \frac{(k+1)}{\langle k_\alpha^{out} \rangle} P_{out}^\alpha(k+1) \int \left[\prod_{i=1}^{k} d\hat{\mu}_i^\alpha \hat{\mathcal{A}}_2^\alpha(\hat{\mu}_i^\alpha) \right] \delta\left(\nu - \frac{1}{1 + \sum_{i=1}^{k} \hat{\mu}_i^\alpha} \right) \tag{31}$$

$$\mathcal{A}_2^\alpha(\mu) = \sum_{k=1}^{\infty} \frac{1}{w_2^\alpha} \sum_{m=k}^{\infty} \frac{(m+1)}{\langle k_\alpha^{out} \rangle} P_{out}^\alpha(m+1) \binom{m}{k} (\hat{w}_1^\alpha)^k (1 - \hat{w}_1^\alpha)^{m-k} \times \int \left[\prod_{i=1}^{k} d\hat{\nu}_i^\alpha \hat{\mathcal{A}}_1^\alpha(\hat{\nu}_i^\alpha) \right] \delta\left(\mu - \frac{1}{\sum_{i=1}^{k} \hat{\nu}_i^\alpha} \right) \tag{32}$$

$$\mathcal{A}_3^\alpha(\gamma) = \sum_{k=1}^{\infty} \sum_{m=k}^{\infty} \frac{1}{w_3^\alpha} \frac{(m+1)}{\langle k_\alpha^{out} \rangle} P_{out}^\alpha(m+1) \binom{m}{k} (\hat{w}_3^\alpha)^k (\hat{w}_2^\alpha)^{m-k} \times \int \left[\prod_{i=1}^{k} d\hat{\gamma}_i^\alpha \hat{\mathcal{A}}_3^\alpha(\hat{\gamma}_i^\alpha) \right] \delta\left(\gamma - \frac{1}{\sum_{i=1}^{k} \hat{\gamma}_i^\alpha} \right) \tag{33}$$

$$\hat{\mathcal{A}}_1^A(\hat{\nu}) = \sum_{k^A=0}^{\infty} \frac{(k^A+1)}{\langle k_A^{in} \rangle} P_{in}^A(k^A+1) (w_2^A)^{k^A} \sum_{k^B=1}^{\infty} \sum_{m^B=k^B}^{\infty} P_{in}^B(m^B) \binom{m^B}{k^B} (w_1^B)^{k^B} (1 - w_1^B)^{m^B - k^B} \int \left[\prod_{i=1}^{k^A} d\mu_i^A \mathcal{A}_2^A(\mu_i^A) \prod_{i=1}^{k^B} d\nu_i^B \mathcal{A}_1^B(\nu_i^B) \right] \times \delta\left(\hat{\nu}^A - \frac{1}{\frac{1}{\sum_{i=1}^{k^B} \nu_i^B} + \sum_{i=1}^{k^A} \mu_i^A} \right) \tag{34}$$

$$\hat{\mathcal{A}}_2^A(\hat{\mu}) = \frac{1}{\hat{w}_2^A} G_1^{A,in}(1-w_1^A) \sum_{k^B} P_{in}^B(k^B)(w_2^B)^{k^B} \int \left[\prod_{i=1}^{k^B} d\mu_i^B \mathcal{A}_2^B(\mu_i^B)\right] \delta\left(\hat{\mu}^A - \sum_{i=1}^{k^B} \mu_i^B\right)$$
$$+ \frac{1}{\hat{w}_2^A}(1-G_0^{B,in}(w_2^B)) \sum_{k^A=1}^{\infty} \sum_{m^A=k^A}^{\infty} \frac{(m+1)}{\langle k_A^{in}\rangle} P_{in}^A(m^A+1) \binom{m^A}{k^A} (w_1^A)^k (1-w_1^A)^{m^A-k^A}$$
$$\times \int \left[\prod_{i=1}^{k^A} d\nu_i^A \mathcal{A}_1(\nu_i^A)\right] \delta\left(\hat{\mu}^A - \frac{1}{\sum_{i=1}^{k^A} \nu_i^A}\right)$$
$$+ \frac{1}{\hat{w}_2^A} \sum_{k^A=1}^{\infty} \sum_{m^A=k^A}^{\infty} \frac{(m+1)}{\langle k_A^{in}\rangle} P_{in}^A(m^A+1) \binom{m^A}{k^A} (w_1^A)^k (1-w_1^A)^{m^A-k^A} \sum_{k^B} P_{in}^B(k^B)(w_2^B)^{k^B}$$
$$\times \int \left[\prod_{i=1}^{k^A} d\nu_i^A \mathcal{A}_1^A(\nu_i^A) \prod_{i=1}^{k^B} d\mu_i^B \mathcal{A}_2^B(\mu_i^B)\right] \delta\left(\hat{\mu}^A - \frac{1}{\frac{1}{\sum_{i=1}^{k^B}\mu_i^B} + \sum_{i=1}^{k^A} \nu_i^A}\right) \tag{35}$$

$$\hat{\mathcal{A}}_3^A(\hat{\gamma}) = \frac{1}{\hat{w}_3^A} G_1^{A,in}(w_2^A) \sum_{k^B=1}^{\infty} \sum_{m^B=k^B}^{\infty} P_{in}^B(k^B) \binom{m^B}{k^B} (w_3^B)^k (w_2^B)^{m^B-k^B}$$
$$\times \int \left[\prod_{i=1}^{k^B} d\gamma_i^B \mathcal{A}_3^B(\gamma_i^B)\right] \delta\left(\gamma^A - \sum_{i=1}^{k_B} \gamma_i^B\right)$$
$$+ \frac{1}{\hat{w}_3^A} \sum_{k^A=1}^{\infty} \sum_{m^A=k^A}^{\infty} \frac{(m^A+1)}{\langle k_A^{in}\rangle} P_{in}^A(m^A+1) \binom{m^A}{k^A} (w_3^A)^k (w_2^A)^{m^A-k^A}$$
$$\times \sum_{k^B=1}^{\infty} \sum_{m^B=k^B}^{\infty} P_{in}^B(k^B) \binom{m^B}{k^B} (w_3^B)^k (w_2^B)^{m^B-k^B} \int \left[\prod_{i=1}^{k^A} d\gamma_i^A \mathcal{A}_3^A(\hat{\gamma}_i^A) \prod_{i=1}^{k^B} d\gamma_i^B \mathcal{A}_3^B(\gamma_i^B)\right]$$
$$\times \delta\left(\gamma^A - \frac{1}{\frac{1}{\sum_{i=1}^{k_B}\gamma_i^B} + \sum_{i=1}^{k^A} \gamma_i^A}\right)$$
$$+ \frac{1}{\hat{w}_3^A}(1-G_0^{B,in}(1-w_1^B)) \sum_{k^A=1}^{\infty} \sum_{m^A=k^A}^{\infty} \frac{(m^A+1)}{\langle k_A^{in}\rangle} P_{in}^A(m^A+1) \binom{m^A}{k^A} (w_3^A)^k (w_2^A)^{m^A-k^A}$$
$$\times \int \left[\prod_{i=1}^{k^A} d\gamma_i^A \mathcal{A}_3^A(\hat{\gamma}_i^A)\right] \delta\left(\gamma^A - \frac{1}{\sum_{i=1}^{k^A} \gamma_i^A}\right) \tag{36}$$

The free energy density $f(\beta) = F(\beta)/N = e_0 - \frac{s_0}{\beta} + \mathcal{O}(1/\beta^2)$ with

$$s_0 = s_{0,a,A} + s_{0,a,B} + s_{0,b} + s_{0,c,A} + s_{0,c,B} \tag{37}$$

where $s_{0,\ell}$ are given by

$$s_{0,a,\alpha} = \sum_{k=1}^{\infty} \sum_{m=k}^{\infty} \binom{m}{k} (\hat{w}_1^\alpha)^k (1-(\hat{w}_1^\alpha))^{m-k} P_{out}^\alpha(m) \overline{\ln \sum_{i=1}^{k} \hat{\nu}_i}$$
$$+ \sum_k P_{out}^\alpha(k)(\hat{w}_2^\alpha)^k \overline{\ln\left(1+\sum_{i=1}^{k} \hat{\mu}_i\right)}$$
$$+ \sum_{k=1}^{\infty} \sum_{m=k}^{\infty} \binom{m}{k} (\hat{w}_3^\alpha)^k (\hat{w}_2^\alpha)^{m-k} P_{out}^\alpha(m) \overline{\ln\left(\sum_{i=1}^{k} \hat{\gamma}_i\right)} \tag{38}$$

$$
\begin{aligned}
s_{0,b} &= (1-G_0^{B,in}(w_2^B))\sum_{k^A=1}^{\infty}\sum_{m^A=k^A}^{\infty}\binom{m^A}{k^A}(w_1^A)^{k^A}(1-(w_1^A))^{m^A-k^A}P_{in}^A(m^A)\overline{\ln\sum_{i=1}^{k^A}\nu_i^A} \\
&+ (1-G_0^{A,in}(w_2^A))\sum_{k^B=1}^{\infty}\sum_{m^B=k^B}^{\infty}\binom{m^B}{k^B}(w_1^B)^{k^B}(1-(w_1^B))^{m^B-k^B}P_{in}^B(m^B)\overline{\ln\sum_{i=1}^{k^B}\nu_i^B} \\
&+ (1-G_0^{B,in}(1-w_1^B))\sum_{k^A=1}^{\infty}\sum_{m^A=k^A}^{\infty}\binom{m^A}{k^A}(w_3^A)^{k^A}(w_2^A)^{m^A-k^A}P_{in}^A(m^A)\overline{\ln\sum_{i=1}^{k^A}\gamma_i^A} \\
&+ (1-G_0^{A,in}(1-w_1^A))\sum_{k^B=1}^{\infty}\sum_{m^B=k^B}^{\infty}\binom{m^B}{k^B}(w_3^B)^{k^B}(w_2^B)^{m^B-k^B}P_{in}^B(m^B)\overline{\ln\sum_{i=1}^{k^B}\gamma_i^B} \\
&+ \Bigg[\sum_{k^B}P_{in}^B(k^B)(w_2^B)^{k^B}\sum_{k^A=1}^{\infty}\sum_{m^A=k^A}^{\infty}\binom{m^A}{k^A}(w_1^A)^{k^A}(1-(w_1^A))^{m^A-k^A}P_{in}^A(m^A) \\
&\quad\times\overline{\ln\left(1+\sum_{i=1}^{k^A}\nu_i^A\sum_{i=1}^{k^B}\mu_i^B\right)}\Bigg] \\
&+ \Bigg[\sum_{k^A}P_{in}^A(k^A)(w_2^A)^{k^A}\sum_{k^B=1}^{\infty}\sum_{m^B=k^B}^{\infty}\binom{m^B}{k^B}(w_1^B)^{m^B}(1-(w_1^B))^{m^B-k^B}P_{in}^B(m^B) \\
&\quad\times\overline{\ln\left(1+\sum_{i=1}^{k^B}\nu_i^B\sum_{i=1}^{k^A}\mu_i^A\right)}\Bigg] \\
&+ \Bigg[\sum_{k^A=1}^{\infty}\sum_{m^A=k^A}^{\infty}\binom{m^A}{k^A}(w_3^A)^{k^A}(w_2^A)^{m^A-k^A}P_{in}^A(m^A) \\
&\quad\times\sum_{k^B=1}^{\infty}\sum_{m^B=k^B}^{\infty}\binom{m^B}{k^B}(w_3^B)^{k^B}(w_2^B)^{m^B-k^B}P_{in}^B(m^B)\overline{\ln\left(1+\sum_{i=1}^{k^A}\gamma_i^A\sum_{i=1}^{k^B}\gamma_i^B\right)}\Bigg]
\end{aligned}
\tag{39}
$$

$$
\begin{aligned}
s_{0,c,\alpha} &= -\langle k^\alpha\rangle_{in}\left\{\hat{w}_1^\alpha(w_1^\alpha+w_3^\alpha)\overline{\ln\hat{\nu}^\alpha}+w_1^\alpha(\hat{w}_1^\alpha+\hat{w}_3^\alpha)\overline{\ln\nu^\alpha}\right. \\
&+ \hat{w}_1^\alpha w_2^\alpha\overline{\ln(1+\hat{\nu}\mu)}+w_1^\alpha\hat{w}_2^\alpha\overline{\ln(1+\nu\hat{\mu})} \\
&+ \left.\hat{w}_1^\alpha w_3^\alpha\overline{\ln\gamma}+w_1^\alpha\hat{w}_3^\alpha\overline{\ln\hat{\gamma}}+w_3^\alpha\hat{w}_3^\alpha\overline{\ln(1+\hat{\gamma}\gamma)}\right\}.
\end{aligned}
\tag{40}
$$

D. Phase transition in the controllability of Poisson duplex networks.

Here we consider the case of two Poisson networks with the same in/out average degree. In other words, we consider the situation in which $\langle k^{A,in}\rangle = \langle k^{A,out}\rangle = \langle k^{B,in}\rangle = \langle k^{B,out}\rangle = c$. The fraction n_D of nodes that are driver nodes of this duplex, is always larger than the double of the fraction of driver nodes in each of the layers taken in isolation (see Fig. 1). Moreover we observe that there is a phase transition in the controllability of these duplex networks, indicated by a discontinuity of n_D for $c = c^\star = 3.22233\ldots$ (see Fig. 1). In order to derive these results, we assumed $w_i^A = w_i^B$ for $i = 1,2,3$ and $\hat{w}_i^A = \hat{w}_i^B$ for $i = 1,2,3$. Therefore the zero-temperature BP equations at the ensemble

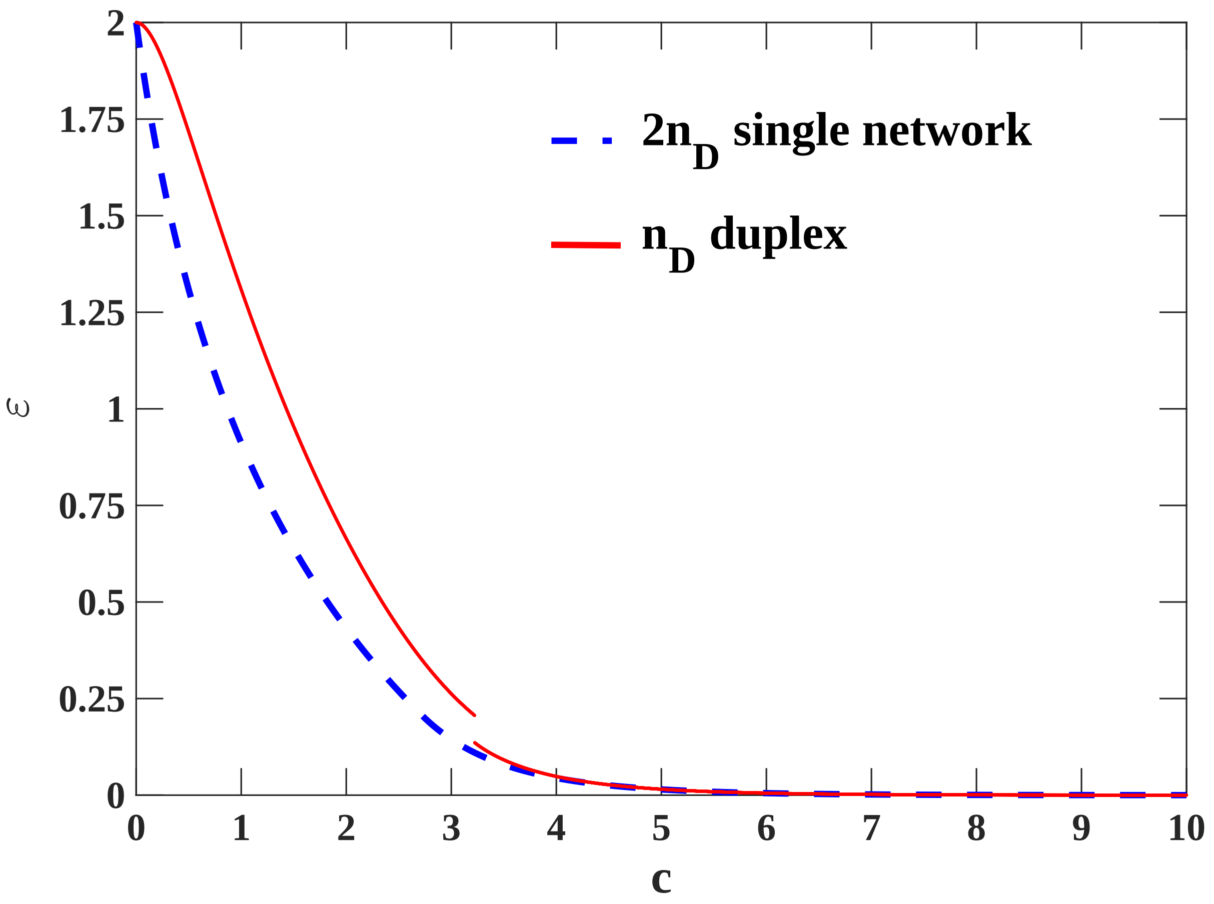

FIG. 1: Density of driver nodes $\varepsilon = n_D$ for a duplex network composed by two Poisson networks with $\langle k^{A,in}\rangle = \langle k^{A,out}\rangle = \langle k^{B,in}\rangle = \langle k^{B,out}\rangle = c$ is indicated with a solid red line and it clearly shows a phase transition for $c = 3.22233\ldots$. In the dashed blue line we display the double of the number of driver nodes $\varepsilon = 2n_D$ for a single Poisson network with the same average degree c, indicating the fraction of driver nodes necessary to control separately the two layers.

level (20) read,

$$\begin{aligned}
w_1 &= e^{-c(1-\hat{w}_2)},\\
w_2 &= \left[1 - e^{-c\hat{w}_1}\right],\\
w_3 &= 1 - w_1 - w_2,\\
\hat{w}_3 &= 1 - \hat{w}_1 - \hat{w}_2,\\
\hat{w}_1 &= e^{-c(1-w_2)}\left[1 - e^{-cw_1}\right],\\
\hat{w}_2 &= \left[1 - e^{-cw_1} + e^{-cw_1}e^{-c(1-w_2)}\right].
\end{aligned} \tag{41}$$

The energy E is given in this case by

$$E = 2\left[e^{-c(1-\hat{w}_2)} - 1 + e^{-c\hat{w}_1}\right] - 2[1 - e^{-cw_1}][1 - e^{-c(1-w_2)}] + 2c\left[\hat{w}_1(1-w_2) + w_1(1-\hat{w}_2)\right]. \tag{42}$$

We notice that the equations for $\hat{w}_1$ and $\hat{w}_2$ can be rewritten to form a closed subsystem of equations,

$$\hat{w}_1 = h_1(\hat{w}_1, \hat{w}_2) = e^{-ce^{-c\hat{w}_1}}\left[1 - e^{-ce^{-c(1-\hat{w}_2)}}\right] \tag{43a}$$

$$\hat{w}_2 = h_2(\hat{w}_1, \hat{w}_2) = \left[1 - e^{-ce^{-c(1-\hat{w}_2)}} + e^{-ce^{-c(1-\hat{w}_2)}}e^{-ce^{-c\hat{w}_1}}\right] \tag{43b}$$

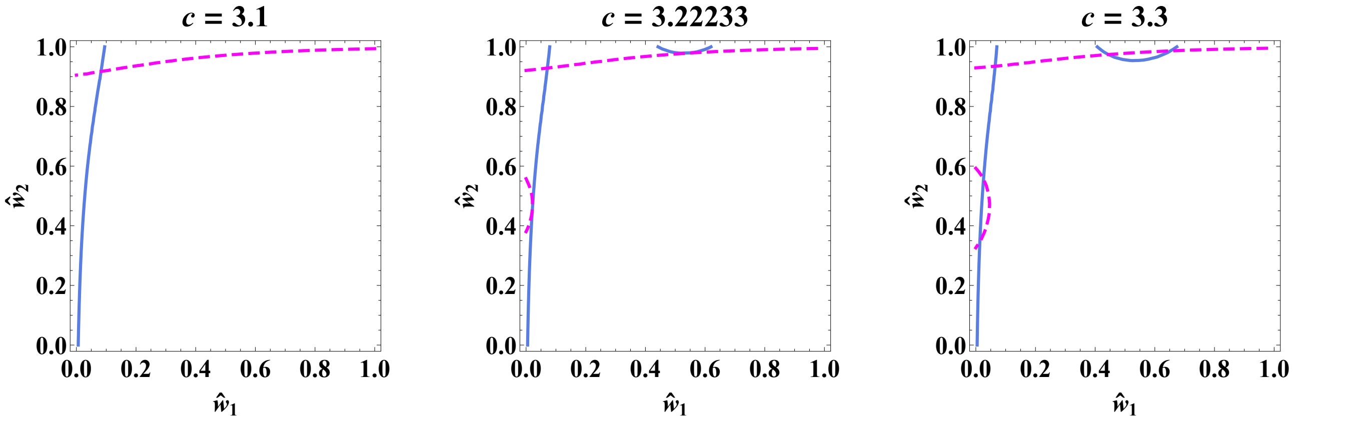

FIG. 2: Plots of the functions $\hat{w}_1 = h_1(\hat{w}_1, \hat{w}_2)$ and $\hat{w}_2 = h_2(\hat{w}_1, \hat{w}_2)$ given by Eqs. $(43a) - (43b)$. The solution of the system of these two equations, corresponds to a crossing of the two curves. We show the emergence of two new solutions of this system of equations for $c > c^{\star} = 3.22233\ldots$. The critical point $c^{\star}$ characterize an hybrid phase transition in the controllability of the duplex network.

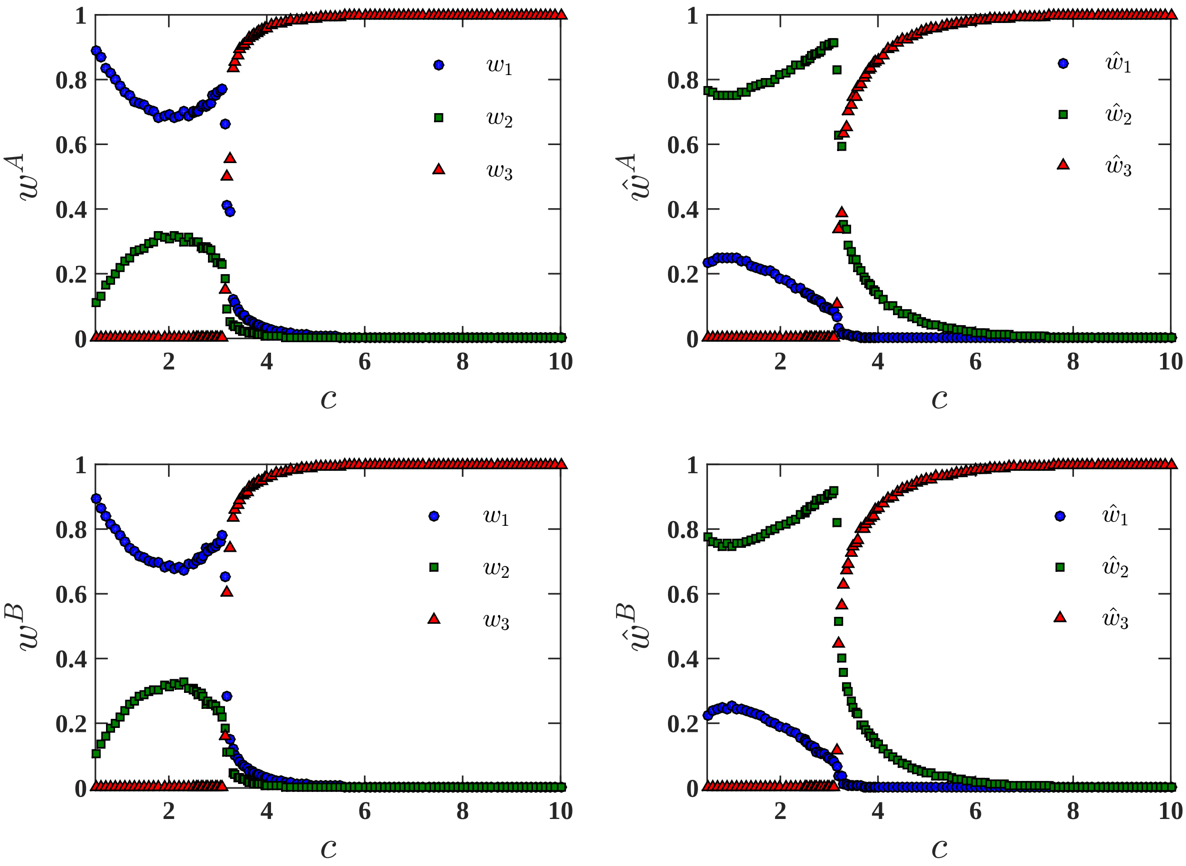

FIG. 3: Values of the probabilities $\{w_i\}_{i=1,2,3}$ and $\hat{w}_{i=1,2,3}$ plotted as a function of the average degree c, for a duplex network formed by two Poisson layers with $\langle k^{A,in} \rangle = \langle k^{A,out} \rangle = \langle k^{B,in} \rangle = \langle k^{B,out} \rangle = c$. These probabilities are calculated directly from BP results obtained over 5 single realizations these multiplex networks with average degree c and $N = 10^4$.

from the solution of which the remaining quantities can be determined.

The value $c^\star$ of the average degree c at which the discontinuity in the number of driver nodes n_D observed in Fig.1 occurs can be found by imposing that the two curves $\hat{w}_1 = h_1(\hat{w}_1, \hat{w}_2)$ and $\hat{w}_2 = h_2(\hat{w}_1, \hat{w}_2)$ of the plane w_1, w_2 for $c = c^\star$ are tangent to each other at their interception. These functions are plotted in Figure 2 where it is possible to observe that for $c > c^\star$ the curves cross in three points while for $c < c^\star$ they cross in one point, and at $c = c^\star$ they are tangent to each other. The critical point $c^\star$ is found by imposing that the Eqs. (43) are satisfied together with the condition

$$|J| = 0, \tag{44}$$

with J indicating the Jacobian of the system of equations $\hat{w}_1 = h_1(\hat{w}_1, \hat{w}_2)$ and $\hat{w}_2 = h_2(\hat{w}_1, \hat{w}_2)$ given by

$$J = \begin{pmatrix} 1 - \frac{\partial h_1(\hat{w}_1,\hat{w}_2)}{\partial \hat{w}_1} & -\frac{\partial h_1(\hat{w}_1,\hat{w}_2)}{\partial \hat{w}_2} \\ -\frac{\partial h_2(\hat{w}_1,\hat{w}_2)}{\partial \hat{w}_1} & 1 - \frac{\partial h_2(\hat{w}_1,\hat{w}_2)}{\partial \hat{w}_2} \end{pmatrix}.$$

Imposing that Eqs. (43) and condition (44) are simultaneously satisfied, the solution $c^\star = 3.222326106\ldots$ is found. For $c < c^\star$ we observe that $w_3 = \hat{w}_3 = 0$. At $c^\star$ we observe a discontinuity in both w_3 and $\hat{w}_3$, but for $c > c^\star$ the functions $h_1(\hat{w}_1, \hat{w}_2)$ and $h_2(\hat{w}_1, \hat{w}_2)$ are analytic, and analyzing Eqs. $(43a) - (43b)$ we obtain the behavior of the order parameters w_3 and $\hat{w}_3$ for $c > c^\star$

$$\begin{aligned} w_3 - w_3^\star &\propto (c - c^\star)^{1/2} \\ \hat{w}_3 - \hat{w}_3^\star &\propto (c - c^\star)^{1/2}, \end{aligned} \tag{45}$$

showing that the transition is hybrid.

We further characterize this phase transition evaluating the number of maximum matchings, i.e. the entropy value of the ground state solutions in the case of two poisson uncorrelated layers. We found that the entropy has a discontinuity at $c^* = 3.22233..$ marking a change in the properties of the solutions.

We further characterize this phase transition evaluating the number of maximum matchings, i.e. the entropy value of the ground state solutions in the case of two poisson uncorrelated layers. The entropy density s follows from Eq. (37) and it is plotted as a function of the average degree c in Fig. 4. The entropy density presents a small jump at $c^* = 3.22233..$ marking a change in the properties of the solutions.

Here we want to modify the degree distribution of the duplex network characterized in this section, by changing the probability of nodes of low degree (degree $0, 1, 2$) that have been shown to be essential to determine the controllability of single layers [5]. Therefore we consider a duplex networks with degree distributions $P_A^{in}(k) = P_A^{out}(k) = P_B^{in}(k) = P_B^{out}(k) = P(k)$ and with minimum degree is 2. In particular we consider $P(k)$ given by

$$P(k) = \begin{cases} 0 & \text{for} \quad k < 2 \\ P(2) & \text{for} \quad k = 2 \\ \kappa \frac{1}{k!} c^k & \text{for} \quad k \in [3, \infty] \end{cases} \tag{46}$$

with κ indicating a normalization constant. In Fig. 5 (on the left) we show the phase diagram of this duplex network described by the dependence of the fraction of driver nodes n_D on c and $P(2)$. The dark grey area defines the region where the zero-energy solution is stable, hence in which to control a duplex one needs only an infinitesimal fraction of driver nodes, i.e. $n_D = 0$. These results are compared with the situation in which the two layers are controlled separately shown in Fig. 5 (on the right). The fraction of driver replica nodes of the duplex network is always larger that the double of the fraction of driver nodes in any single layer taken in isolation. Moreover the region in which the fully controllable solution is stable is the same for the duplex network, and for the single networks in the layers of the duplex network taken in isolation. This result is consistent with the theoretical expectations obtained in Sec. IV B. In fact the in- and out-degree distributions of the two layers are the same.

E. Controllability of scale-free duplex networks

Following Sec. IV D we consider now the case of two uncorrelated layers composed by two power-law networks with $P(k^{A,in}) = P(k^{A,out}) = P(k^{B,in}) = P(k^{B,out}) = P(k) \propto k^{-\gamma}$ and minimal degree $m = 1$. Similarly to the poisson case, the fraction n_D of driver nodes of this duplex, is always larger than the double of the fraction of driver nodes in each of the layers taken in isolation (see Fig. 6).

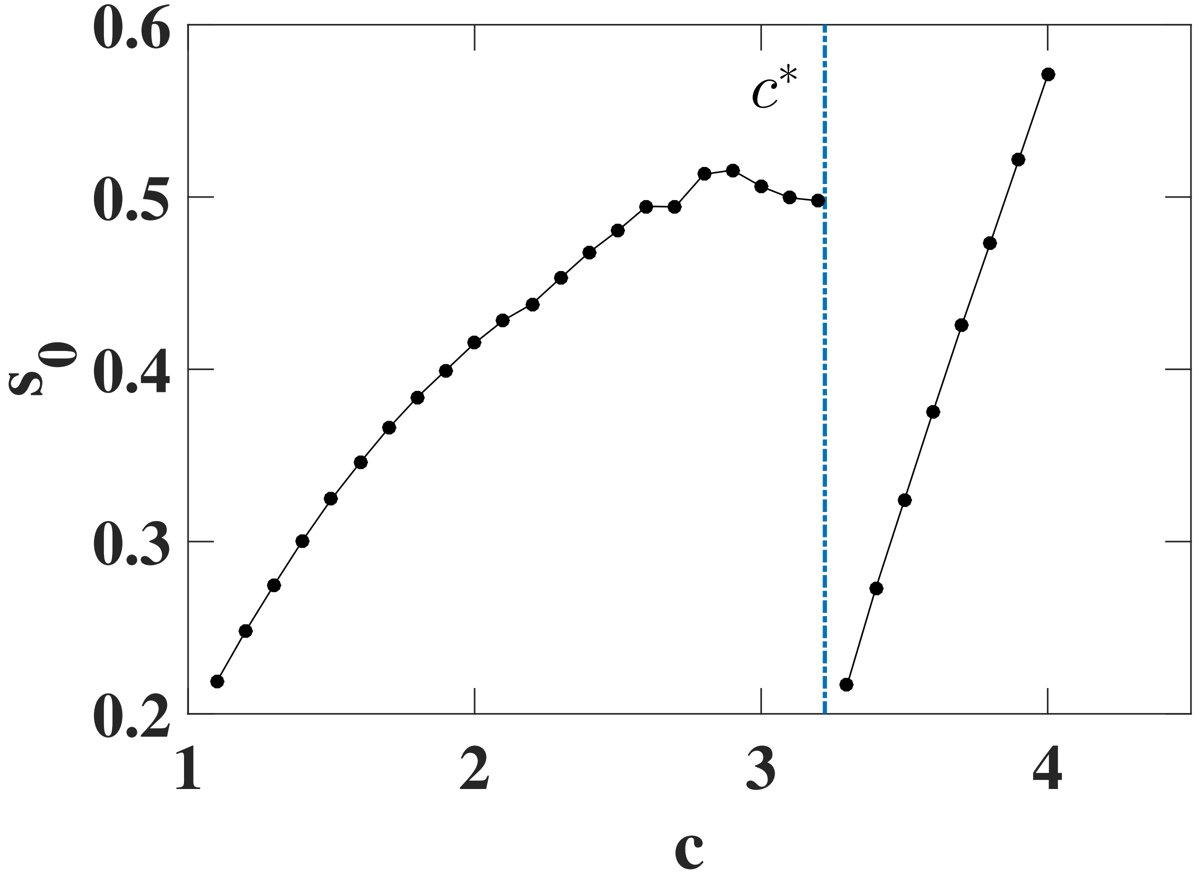

FIG. 4: Entropy density s for a duplex network composed by two Poisson networks with $\langle k^{A,in}\rangle = \langle k^{A,out}\rangle = \langle k^{B,in}\rangle = \langle k^{B,out}\rangle = c$. At $c^* = 3.22233..$, average degree corresponding to the hybrid phase transition, the entropy density s displays a finite jump.

Moreover, low-degree nodes significantly affect the controllability of duplex networks formed by scale-free networks. We consider a duplex network with $P_A^{in}(k) = P_A^{out}(k) = P_B^{in}(k) = P_B^{out}(k) = P(k)$ and $P(k)$ given by

$$P(k) = \begin{cases} 0 & \text{if} \quad k = 1 \\ P(2) & \text{if} \quad k = 2 \\ \kappa k^{-\gamma} & \text{if} \quad k \in [3, M] \end{cases} \tag{47}$$

with κ indicating the normalization sum and $\gamma > 2$. We consider uncorrelated networks, therefore the cutoff M on the degrees of the nodes will be given by

$$M = \min(\sqrt{N}, \{[1 - P(1) - P(2)]N\}^{1/(\gamma-1)}). \tag{48}$$

In other words, the cutoff M is given by the minimum between the structural cutoff of the network and the natural cutoff of the degree distribution. In Fig. 7 (on the left) we present the phase diagram of a duplex network displaying the fraction of driver nodes n_D as a function of the parameters γ and $P(2)$. The dark grey area is associated with the stable zero-energy solution while outside this region, the minimum fraction of driver nodes necessary for a full duplex control follows the colorcode. We compare these results with the situation in which the two layers are controlled separately (on the right). We observe that the number of driver replica nodes in the duplex is always greater than the total number of driver nodes of the single layer taken in isolation, provided that the duplex network is not fully controllable. We note that for the degree distribution considered in this case, consistently with the theoretical results obtained in Sec. $IV\,B$, we observe that the region for the stability of the full controllability solution for the duplex network is the same of the region for the stability of the full controllability solution in the single layers.

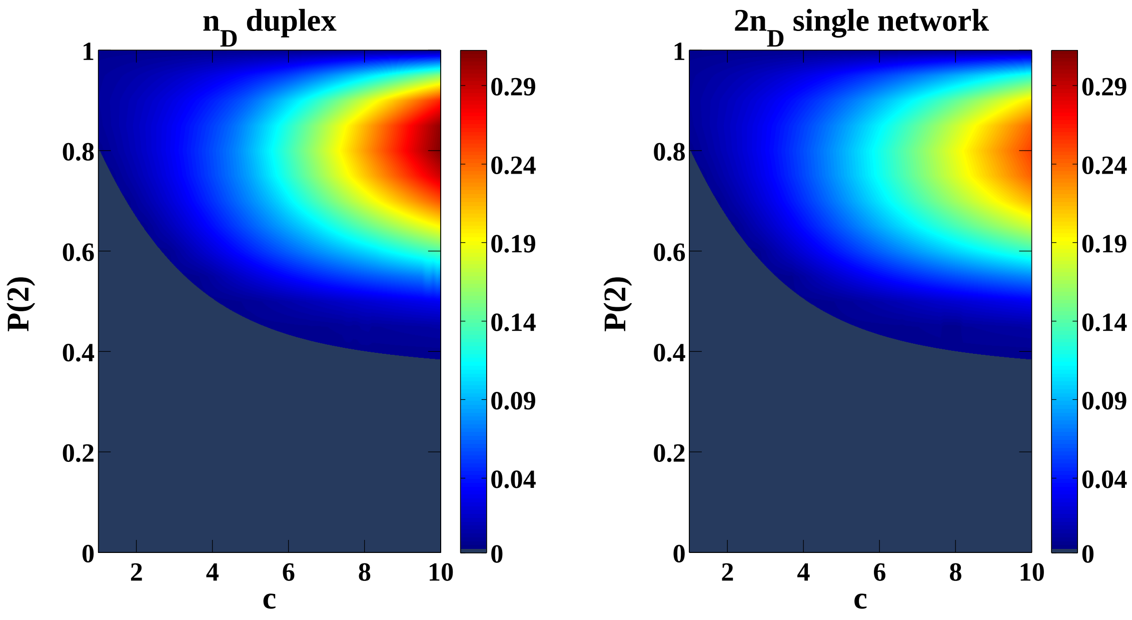

FIG. 5: On the left: the density of driver nodes n_D as a function of the parameters c and $P(2)$ is plotted for duplex networks with $P_A^{in}(k) = P_A^{out}(k) = P_B^{in}(k) = P_B^{out}(k) = P(k)$ and $P(k)$ given by Eq. (46). On the right: the double of the density of driver nodes n_D for single layers with degree distribution $P^{in}(k) = P^{out}(k) = P(k)$ and $P(k)$ given by (46) is plotted as a function of c and $P(2)$.

Finally , in Fig. 8 we compare our theoretical results for the ensemble of duplex networks with degree distributions $P_A^{in}(k) = P_A^{out}(k) = P_B^{in}(k) = P_B^{out}(k) = P(k)$ and $P(k)$ given by Eq. (47), with those obtained by the message-passing (BP) algorithm, finding a good agreement (Eq. 29 returns a limit value for $P(2)$ equal to 0.181947).

V. EFFECT OF DEGREE CORRELATIONS ON CONTROLLABILITY OF MULTIPLEX NETWORKS

In order to analyze the effect of degree correlations [1] on the controllability of multiplex networks, we correlate the degree of the replica nodes in the two layers of a duplex network formed by layer A and layer B. In particular we consider two cases: a duplex network in which only the low in-degree nodes (nodes of in-degree $0, 1, 2$) are correlated and a duplex networks in which the in-degrees of the replica nodes are correlated independently on their value. For each case, we define the joint in-degree distribution $P^{in}(k^A, k^B)$ between layers and the corresponding expression of the zero-temperature BP equations in the correlated ensemble of networks.

In the first case we consider a joint in-degree distribution $P^{in}(k^A, k^B)$ given by

$$P_{in}(k^A, k^B) = \begin{cases} p\delta_{k^B,k^A}P(k^A) + (1-p)P(k^A)P(k^B), & \text{for} \quad k^A \leq 2 \\ (1-p)P(k^A)P(k^B), & \text{for} \quad k^A > 2 \quad k^B \leq 2 \\ p\frac{P(k^B)}{C}P(k^A) + (1-p)P(k^A)P(k^B), & \text{for} \quad k^A > 2 \quad k^B > 2 \end{cases},$$

where $C = 1 - \sum_{k\leq 2} P(k)$ where $P(k)$ is a given normalized degree distribution. The distributions of the fields over the links of this ensemble of networks are given by

$$\begin{aligned} \mathcal{P}_\alpha(h^\alpha) &= w_1^\alpha \delta(h^\alpha - 1) + w_2^\alpha \delta(h^\alpha + 1) + w_3^\alpha \delta(h^\alpha), \\ \hat{\mathcal{P}}_\alpha(\hat{h}^\alpha) &= \hat{w}_1^\alpha \delta(\hat{h}^\alpha - 1) + \hat{w}_2^\alpha \delta(\hat{h}^\alpha + 1) + \hat{w}_3^\alpha \delta(\hat{h}^\alpha), \end{aligned} \tag{49}$$

where $\alpha = A, B$ and where the probabilities $w_1^\alpha, w_2^\alpha, w_3^\alpha$ are normalized $w_1^\alpha + w_2^\alpha + w_3^\alpha = 1$ as well as the probabilities $\hat{w}_1^\alpha, \hat{w}_2^\alpha, \hat{w}_3^\alpha$ that satisfy the equation $\hat{w}_1^\alpha + \hat{w}_2^\alpha + \hat{w}_3^\alpha = 1$. The zero-temperature BP (Max-Sum) equations (16) averaged

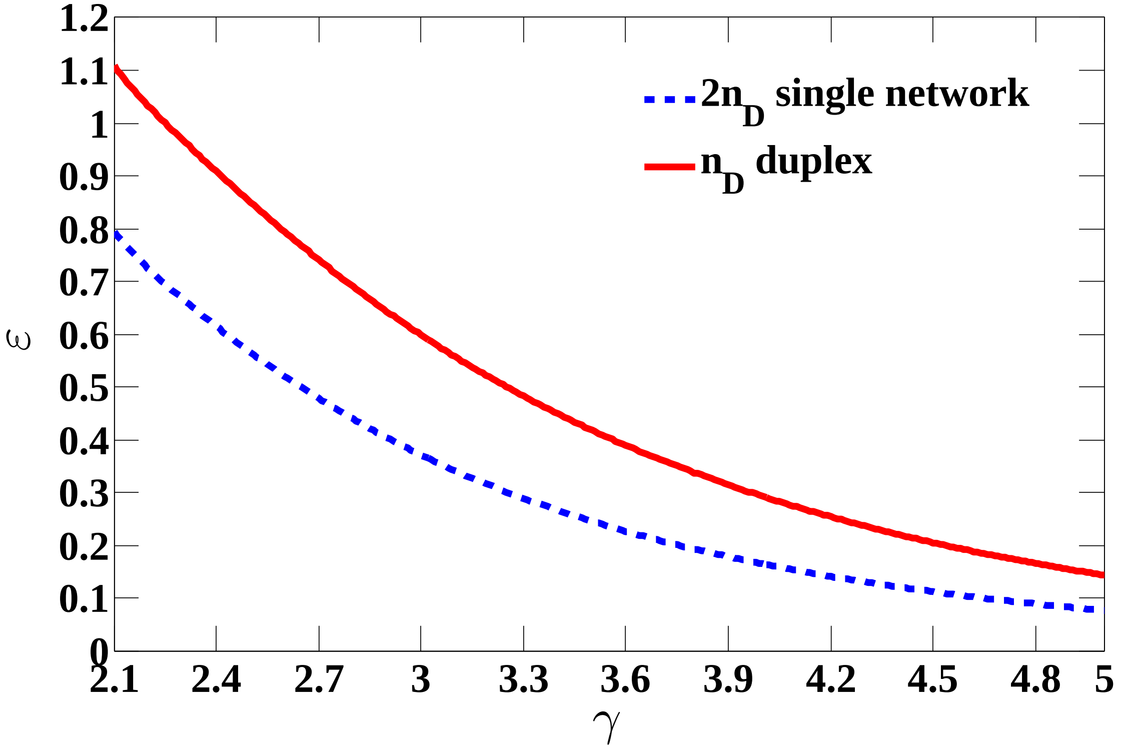

FIG. 6: Density of driver nodes $\varepsilon = n_D$ for a duplex network composed by two power-law networks with $P(k^{A,in}) = P(k^{A,out}) = P(k^{B,in}) = P(k^{B,out}) = P(k) \propto k^{-\gamma}$ and minimal degree $m = 1$ as a function of γ (indicated with a solid red line). The minimum in/out degree 1 and the maximum in/out degree is given by the structural cutoff with $N = 10^4$. In the dashed blue line we display the double of the number of driver nodes $\varepsilon = 2n_D$ for a single power-law network with the same in/out degree distributions $P(k)$, indicating the fraction of driver nodes necessary to control separately the two layers.

over this ensemble of networks can be expressed in terms of the probabilities $\{w_i^{\alpha}\}_{i=1,2,3}$ and $\{w_i^{\alpha}\}_{i=1,2,3}$ as

$$\begin{aligned}
\hat{w}_1 &= p\left[\frac{P(1)}{\langle k\rangle}w_1 + \frac{2P(2)}{\langle k\rangle}w_2(1-(1-w_1)^2) + (G_1(w_2) - \frac{P(1)}{\langle k\rangle} - \frac{2P(2)}{\langle k\rangle}w_2)(1-\tilde{G}_0(1-w_1))\right] \\
&\quad + (1-p)G_1(w_2)\left[1-G_0(1-w_1)\right] \qquad (50a) \\
\hat{w}_2 &= p\left[\frac{P(1)}{\langle k\rangle}w_2 + \frac{2P(2)}{\langle k\rangle}(w_1 + w_2^2(1-w1)) + 1 - \frac{P(1)}{\langle k\rangle} - \frac{2P(2)}{\langle k\rangle}\right. \\
&\quad \left. -(G_1(1-w_1) - \frac{P(1)}{\langle k\rangle} - \frac{2P(2)}{\langle k\rangle}(1-w_1))(1-\tilde{G}_0(w_2))\right] \\
&\quad + (1-p)\left[1 - G_1(1-w_1) + G_1(1-w_1)G_0\left(w_2\right)\right] \qquad (50b)
\end{aligned}$$

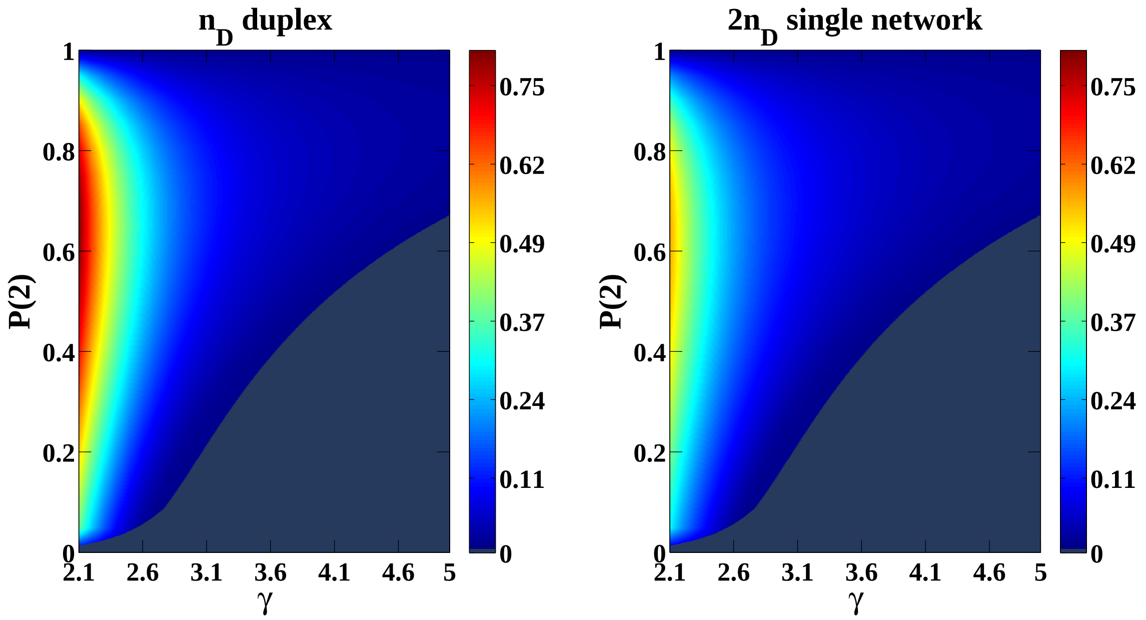

FIG. 7: On the left: the density of driver nodes n_D as a function of the parameters γ and $P(2)$ for duplex networks of $N = 10^6$ nodes with degree distributions $P_A^{in}(k) = P_A^{out}(k) = P_B^{in}(k) = P_B^{out}(k) = P(k)$ and $P(k)$ given by Eq. (47). On the right: double of the density of driver nodes n_D as a function of the parameters γ and $P(2)$ for single networks of $N = 10^6$ nodes with degree distributions $P^{in}(k) = P^{out}(k) = P(k)$ and $P(k)$ given by Eq. (47).

where

$$\begin{aligned} G_0(z) &= \sum_k P(k) z^k \\ G_1(z) &= \sum_k \frac{k}{\langle k \rangle} P(k) z^k \\ \tilde{G}_0(z) &= \sum_{k \geq 3} \frac{P(k)}{C} z^k. \end{aligned} \tag{51}$$

Finally the energy E is given by

$$\begin{aligned} E &= 2\left\{G_0\left(\hat{w}_2\right) - \left[1 - G_0(1-\hat{w}_1)\right]\right\} + 2\langle k \rangle \left[\hat{w}_1(1-w_2) + w_1(1-\hat{w}_2)\right] - 2(1-p)\left\{[1 - G_0(1-w_1)][1 - G_0(w_2)]\right\} \\ &- 2p\left\{P(1)w_1(1-w_2) + P(2)(1-(1-w_1)^2)(1-w_2^2) + C(1-G_0^t(1-w_1))(1-\tilde{G}_0(w_2))\right\} \end{aligned} \tag{52}$$

In the second case we consider the joint degree distribution $P^{in}(k^A, k^B)$ given by

$$P^{in}(k^A, k^B) = p\delta_{k^B,k^A} P(k^A) + (1-p)P(k^A)P(k^B), \tag{53}$$

where $P(k)$ is a given normalized degree distribution. The distributions of the fields over the links of this ensemble of duplex networks are given by

$$\begin{aligned} \mathcal{P}_\alpha(h^\alpha) &= w_1^\alpha \delta(h^\alpha - 1) + w_2^\alpha \delta(h^\alpha + 1) + w_3^\alpha \delta(h^\alpha), \\ \hat{\mathcal{P}}_\alpha(\hat{h}^\alpha) &= \hat{w}_1^\alpha \delta(\hat{h}^\alpha - 1) + \hat{w}_2^\alpha \delta(\hat{h}^\alpha + 1) + \hat{w}_3^\alpha \delta(\hat{h}^\alpha), \end{aligned} \tag{54}$$

where $\alpha = A, B$ and where the probabilities $w_1^\alpha, w_2^\alpha, w_3^\alpha$ are normalized $w_1^\alpha + w_2^\alpha + w_3^\alpha = 1$ as well as the probabilities $\hat{w}_1^\alpha, \hat{w}_2^\alpha, \hat{w}_3^\alpha$ that satisfy the equation $\hat{w}_1^\alpha + \hat{w}_2^\alpha + \hat{w}_3^\alpha = 1$. We get the equations

$$\hat{w}_1 = p\left[G_1(w_2) - (1-w_1)G_1(w_2(1-w_1))\right] + (1-p)G_1(w_2)\left[1 - G_0(1-w_1)\right] \tag{55a}$$

$$\hat{w}_2 = p\left[1 - G_1(1-w_1) + w_2 G_1(w_2(1-w_1))\right] + (1-p)\left[1 - G_1(1-w_1) + G_1(1-w_1)G_0\left(w_2\right)\right], \tag{55b}$$

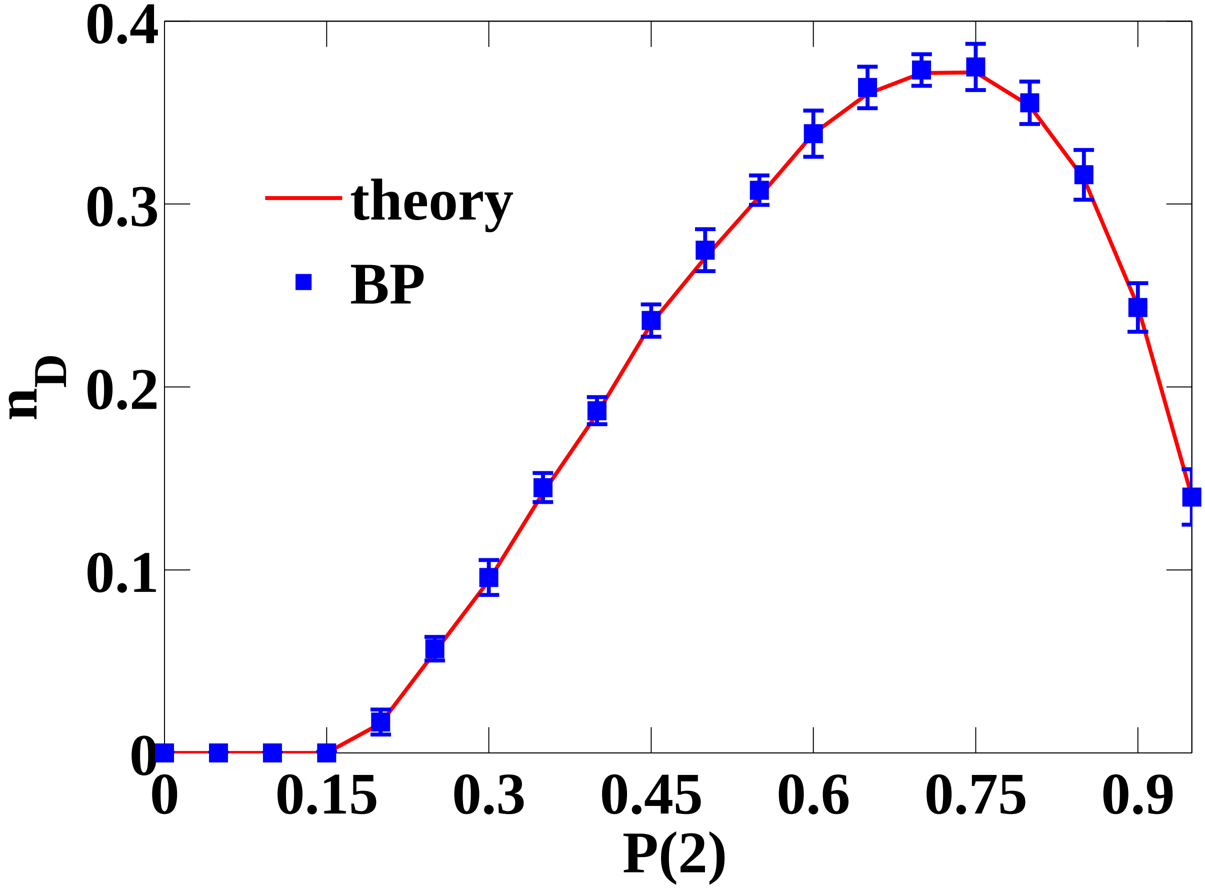

FIG. 8: Density of driver nodes n_D as a function of $P(2)$ for a duplex network with $P_A^{in}(k) = P_A^{out}(k) = P_B^{in}(k) = P_B^{out}(k) = P(k)$, $P(k)$ given by Eq. (47) and $\gamma = 2.3$. The fraction of driver nodes computed with the zero-temperature BP (Max-Sum) algorithm on a duplex network of $N = 10^4$ nodes (averaged over 25 network realizations) is compared with the theoretical expectation for the density n_D in an ensemble of random duplex networks with the given degree distributions.

where the generating functions $G_0(z)$ and $G_1(z)$ are defined as

$$G_0(z) = \sum_k P(k) z^k,$$
$$G_1(z) = \sum_k \frac{k}{\langle k \rangle} P(k) z^k. \tag{56}$$

The energy E in this ensemble is given by

$$\begin{aligned} E &= 2\left\{G_0\left(\hat{w}_2\right) - \left[1 - G_0(1-\hat{w}_1)\right]\right\} + 2\langle k\rangle\left[\hat{w}_1(1-w_2) + w_1(1-\hat{w}_2)\right] \\ &- 2(1-p)\left\{\left[1-G_0(1-w_1)\right]\left[1-G_0(w_2)\right]\right\} - 2p\left\{1 - G_0(1-w_1) - G_0(w_2) + G_0(w_2(1-w_1))\right\} \end{aligned} \tag{57}$$

The degree correlation of low in-degree nodes can modify the number of driver nodes n_D found in duplex networks (see Fig. 9 for the case of a duplex network formed by Poisson layers with $\left\langle k^{A,in}\right\rangle = \left\langle k^{A,out}\right\rangle = \left\langle k^{B,in}\right\rangle = \left\langle k^{B,out}\right\rangle = c$). Once the low in-degree nodes are correlated, correlating also the other in-degrees of the network does not change substantially the number of driver nodes n_D as discussed in the main body of the paper.

---

[1] S. Boccaletti, G. Bianconi, R. Criado, C.I. del Genio, J. Gómez-Gardeñes, M. Romance, I. Sendiña-Nadal, Z. Wang, M. Zanin, Physics Reports **544**, 1 (2014).

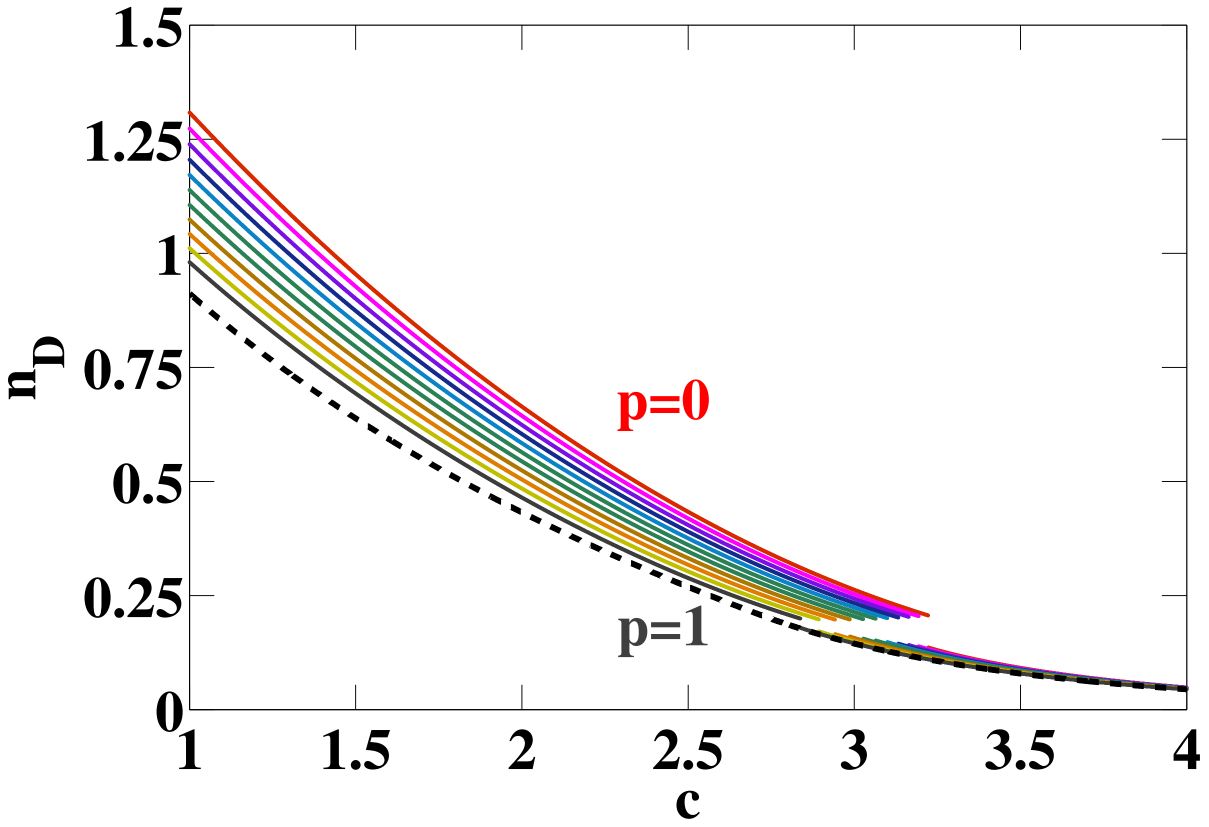

FIG. 9: The density of the driver nodes n_D in a duplex network formed by two Poisson networks with $\langle k^{A,in}\rangle = \langle k^{A,out}\rangle = \langle k^{B,in}\rangle = \langle k^{B,out}\rangle = c$ and with correlated low in-degrees is plotted as function of c for different values of p. The result for the two separate layers is shown in black (dashed curve) while the situation for uncorrelated layers is shown in red. The value of p increases going from the red curve ($p = 0$) to the grey curve ($p = 1$).

[2] J.-J. Slotine ,W. Li W. *Applied Nonlinear Control* (Prentice-Hall, 1991).
[3] C.-T. Lin, IEEE Trans. Auto. Contr. **19**, 201 (1974).
[4] Y.-Y. Liu, J.-J. Slotine, A.-L. Barabási, Nature **473**, 167 (2011).
[5] G. Menichetti, L. Dall'Asta and G. Bianconi, Phys. Rev. Lett. **113**, 078701 (2014).
[6] L. Zdeborová and M. Mézard, J. Stat Mech. P05003 (2006).
[7] F. Altarelli, A. Braunstein, A. Ramezanpour, and R. Zecchina, Phys. Rev. Lett. **106**, 190601 (2011).
[8] M. Mézard, A. Montanari *Information, physics and computation* (Oxford University Press, Oxford, 2009).
[9] M. Mézard, G. Parisi, Eur. Phys. J. B **20**,217 (2001).
[10] O. C.Martin, R. Monasson, and R. Zecchina,Theoretical computer science **265**, 3 (2001).
[11] A. K. Hartmann and M. Weigt, *Phase Transitions in Combinatorial Optimization Problems*, (WILEY-VCH, Weinheim, 2005).
